# Combining controls can improve power in two-stage association studies

**DOI:** 10.1186/s12863-018-0675-y

**Published:** 2018-10-03

**Authors:** James Liley

**Affiliations:** Department of Medicine, University of Cambridge, Addenbrooke’s Hospital, Cambridge, UK

**Keywords:** Case-control study, Replication, GWAS

## Abstract

**Background:**

High dimensional case control studies are ubiquitous in the biological sciences, particularly genomics. To maximise power while constraining cost and to minimise type-1 error rates, researchers typically seek to replicate findings in a second experiment on independent cohorts before proceeding with further analyses. This can be an expensive procedure, particularly when control samples are difficult to recruit or ascertain; for example in inter-disease comparisons, or studies on degenerative diseases.

**Results:**

This paper presents a method in which control (or case) samples from the discovery cohort are re-used in a replication study. The theoretical implications of this method are discussed and simulated genome-wide association study (GWAS) tests are used to compare performance against the standard approach in a range of circumstances.

Using similar methods, a procedure is proposed for ‘partial replication’ using a new independent cohort consisting of only controls. This methods can be used to provide some validation of findings when a full replication procedure is not possible.

The new method has differing sensitivity to confounding in study cohorts compared to the standard procedure, which must be considered in its application. Type-1 error rates in these scenarios are analytically and empirically derived, and an online tool for comparing power and error rates is provided.

**Conclusions:**

In several common study designs, a shared-control method allows a substantial improvement in power while retaining type-1 error rate control. Although careful consideration must be made of all necessary assumptions, this method can enable more efficient use of data in GWAS and other applications.

**Electronic supplementary material:**

The online version of this article (10.1186/s12863-018-0675-y) contains supplementary material, which is available to authorized users.

## Background

High-dimensional case-control studies have become a mainstay of investigation of pathophysiology in complex diseases and traits. An important part of their analysis is the process of replication [1], in which the results of a high-dimensional study are used to inform the design of a second study at a subset of the original variables, with a joint analysis used to determine overall association.

Replicating studies in this way has the advantage of increasing the effective study sample sizes without requiring measurement of all variables in all samples. It also serves to protect against false-positives due to systematic errors in the original datasets, by re-testing association in a second nominally independent dataset.

Replication has a significant cost, and can require large numbers of samples, especially when associated variables have small effects (i.e. [2]). There is therefore a need to minimise the number of additional samples which need to be analysed. This paper presents a method to perform replication by combining controls in both the original ‘discovery’ and second ‘replication’ datasets, potentially reducing the number of new samples required. Shared-control approaches can improve study efficiency in many related applications in which studies are compared [3–8].

Results from original and replication datasets for which some or all controls are shared cannot be directly compared due to the correlation between test statistics directly resulting from shared controls even under the null hypothesis [5]; use of the same thresholds in a shared-control design as used in an independent-controls design will lead to higher type-1 error rates. This paper demonstrates a simple adaptation to a standard design to account for the changed correlation structure and retain control of type-1 error rate, only requiring a change to one *p*-value threshold.

An important purpose of replication is control against false-positives arising from variables for which confounding causes an apparent case-control difference in one of the discovery- or replication- phase experiments, but not the other. The action of sharing control samples results in a different spectrum of sensitivity to variables of this type. It necessitates a sacrifice of type-1 error rate control in variables for which confounding affects the discovery-phase control cohort, but improves type-1 error rate control in variables for which confounding affects the replication-phase control cohort. The type-1 error rate is largely equivalent to an independent-controls design in variables affected by confounding in either case cohort.

The new spectrum of false positive rates can be advantageous in circumstances where control samples in the replication cohort are less well-ascertained than those in the discovery cohort. This may be the case in studies on degenerative disease, where control ascertainment is generally uncertain, and population-sourced controls may be used for replication. The shared-control design can reduce power losses from mis-specified controls in the replication cohort, as well as reducing false-positive rates caused by confounding in the cohort.

When used with shared cases instead of controls, this method can be adapted to a ‘partial replication’ procedure where only a new control set is used. Although not equivalent to a full replication in an independent dataset, the procedure enables improvement in type-1 error rates and control over confounding. This is applicable in studies on rare traits, where all available samples need to be included in the discovery analysis for adequate power.

Throughout this paper, GWAS terminology will be used (single-nucleotide polymorphisms (SNPs), allele frequency, variants etc) although the method is applicable to any high-dimensional case control study. ‘Controls’ will be considered to generally be samples unaffected by a disease or trait of interest, although the method can be applied with case/control labels swapped, or applied to comparisons between subgroups of a case group.

Differences in power (at fixed type-1 error rate) between standard (independent-controls) and new (shared-control) methods are established by considering hypothesis tests typical of those found in GWAS. Asymptotic analytical results are established where possible, but all type 1/type 2 error rates are readily tractable empirically to good accuracy given study sizes and proposed *p*-value thresholds, and a tool is provided to do this at https://wallacegroup-liley.shinyapps.io/replication_shared/.

## Results

### Overview of method

We assume a GWAS dataset of a set of cases *C*_1_ and controls *C*_0_ used in a ‘discovery’ phase of a GWAS or similar study, and corresponding sets of cases and controls $C_{1}^{\prime }$, $C_{0}^{\prime }$ in the replication phase. We assume that *C*_0_ and *C*_1_ are genotyped at a set of SNPs *S* and $C_{0}^{\prime }$, $C_{1}^{\prime }$ at a set *S*^′^⊆*S*.

For each SNP we designate *μ*_1_, *μ*_0_, $\mu _{1}^{\prime }$, $\mu _{0}^{\prime }$ as the population minor allele frequency in the corresponding group, and *m*_1_, *m*_0_, $m_{1}^{\prime }$, $m_{0}^{\prime }$ as the observed allele frequency (so *E*(*m*_*i*_)=*μ*_*i*_). We designate two null hypotheses; $H_{0}^{\cup }:(\mu _{1}=\mu _{0}) \cup (\mu _{1}^{\prime }=\mu _{0}^{\prime })$ and $H_{0}^{=}:(\mu _{1}=\mu _{0} = \mu _{1}^{\prime }=\mu _{0}^{\prime })$, noting that $H_{0}^{\cup } \supseteq H_{0}^{=}$. In a typical conservative GWAS approach, we seek to test against $H_{0}^{\cup }$, since *μ*_1_≠*μ*_0_ or $\mu _{1}^{\prime } \neq \mu _{0}^{\prime }$ may hold at non-disease associated SNPs due to confounding in the original or replication studies respectively. The alternative null hypothesis $(\mu _{1}=\mu _{0} \cap \mu _{1}^{\prime }=\mu _{0}^{\prime })$, which implies $H_{0}^{=}$ and is implied by $H_{0}^{\cup }$, is more appropriate than $H_{0}^{=}$ in cases where replication is performed in a different population than discovery. However, this situation is not adaptable to a shared-control design.

A typical two-stage genetic testing procedure [9], which we will refer to as method A, begins by comparing genotypes of *C*_1_ and *C*_0_ at SNPs *S* generating *p*-values *p*_*d*_ (discovery). A subset *S*^′^ of SNPs reaching putative significance level *p*_*d*_<*α* are genotyped in $C_{0}^{\prime }$ and $C_{1}^{\prime }$, with genotypes compared to generate *p*-values *p*_*r*_ (replication stage). Finally, genotypes are compared between $C_{0} \cup C_{0}^{\prime }$ and $C_{1} \cup C_{1}^{\prime }$ at SNPs *S*^′^ to generate *p*-values *p*_*m*_ (meta-analytic stage). SNPs are designated as ‘hits’ if *p*_*d*_<*α*,*p*_*r*_<*β*,*p*_*m*_<*γ* for some *β*, *γ*, and all effects have the same direction. The values *α*, *β*, *γ* may not be explicitly stated in some study designs, although they are usually implicitly present. This is discussed further in the “Choice of thresholds” section below.

**Fig. 1:**
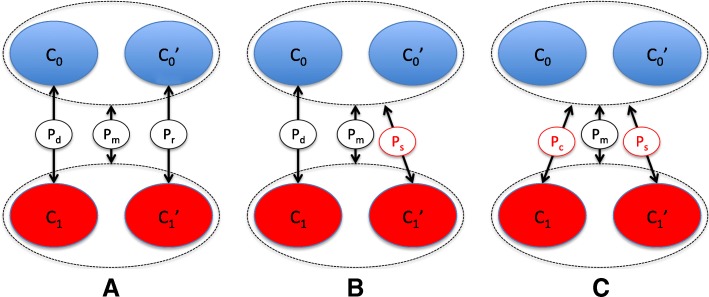
Diagram of methods **a**, **b**, and **c**. Method **b** differs by comparing $C_{1}^{\prime }$ to pooled *C*_0_ and $C_{0}^{\prime }$ at the replication stage to generate *p*-value *P*_*s*_ instead of *P*_*r*_. Method **c** also pools controls at the discovery phase, comparing *C*_1_ to pooled *C*_0_ and $C_{0}^{\prime }$ to generate *p*-values *P*_*c*_ instead of *P*_*d*_. A ‘hit’ is declared in method **a** if *P*_*d*_<*α*, *P*_*r*_<*β*, *P*_*m*_<*γ*, in method **b** if *P*_*d*_<*α*, *P*_*s*_<*β*^∗^, *P*_*m*_<*γ* and in method **c** if *P*_*c*_<*α*, *P*_*s*_<*β*^⊥^, *P*_*m*_<*γ*

The main modification proposed in this paper, denoted as method B, differs at the replication stage in that $C_{1}^{\prime }$ is compared with $C_{0} \cup C_{0}^{\prime }$ at *S*^′^ instead of just *C*_0_ (Fig. 1). The *p*-values resulting from the modified replication stage are termed *p*_*s*_, and the criterion to designate a hit changed to *p*_*d*_<*α*,*p*_*s*_<*β*^∗^,*p*_*m*_<*γ*, with all effects in the same direction. The threshold *β*^∗^ is chosen to conserve type-1 error rate between methods (see “Methods” section and Additional file 1: Appendix 1). This requires estimation of systematic correlation between Z scores, which may be estimated either empirically or (in some cases) analytically.

A second modification, denoted method C, combines *C*_0_ and $C_{0}^{\prime }$ at both the discovery and replication phase (see Fig. 1). This is analogous to a situation in which only a single control cohort is available, and a choice must be made to split it between discovery and replication procedures or to use it for both. In this case, $C_{0} \cup C_{0}^{\prime }$ is compared with *C*_1_ at SNPs *S* in the discovery phase to produce *p*-values *p*_*c*_, then $C_{0} \cup C_{0}^{\prime }$ is compared with $C_{1}^{\prime }$ at SNPs *S*^′^ at the replication phase and compared with $C_{1} \cup C_{1}^{\prime }$ at the meta-analytic stage to produce *p*-values *p*_*s*_ and *p*_*m*_ as before. A hit is determined by *p*_*c*_<*α*,*p*_*s*_<*β*^⊥^,*p*_*m*_<*γ*, with all effects in the same direction. Again, *β*^⊥^ is chosen to maintain the type-1 error rate between methods.

### General properties

For SNPs in $H_{0}^{=}$, the overall type-1 error rate is conserved between methods by the definition of *β*^∗^, *β*^⊥^ (Eq. 4) at a level *P*_0_. It is shown in Additional file 1: Appendix 2.2 that *β*>*β*^∗^>*β*^⊥^. For SNPs in $H_{0}^{\cup } \setminus H_{0}^{=}$ the type-1 error rates differ between methods. Such SNPs may be characterised by the group(s) amongst *C*_0_, *C*_1_, $C_{0}^{\prime }$, $C_{1}^{\prime }$ in which their expected minor allele frequency (MAF) is aberrant from the expected MAF in the population which the group ostensibly represents. ‘Aberrance’ is taken to mean an incorrect expected value from systematic measurement error or uncorrected confounding, rather than random deviance around a correct expected value.

Bounds on type-1 error rates with aberrance in each group are shown in Table 1. Methods B and C necessitate sacrificing bounds on error rates with aberrance in *C*_0_ and $C_{0},C_{0}^{\prime }$ respectively. The bound on error with aberrance in $C_{1}^{\prime }$ improves through methods A-C. In the “Methods” section, it is shown that the type-1 error with aberrance in $C_{0}^{\prime }$ decreases from methods A to B, and the error with aberrance in $C_{1}^{\prime }$ increases from A through C, although the upper bound is the same for both.

**Table 1:** Upper bounds on type 1 error rates with aberrance in cohorts, with *β*>*β*^∗^>*β*^⊥^

	Aberrant				
	None	*C*_0_	$C_{0}^{\prime }$	*C*_1_	$C_{1}^{\prime }$
M. A	*P*_0_	*β*	*α*	*β*	*α*
M. B	*P*_0_	1	*α*	*β* ^∗^	*α*
M. C	*P*_0_	1	1	*β* ^⊥^	*α*

### Bias in effect size estimates

If a set of variants in a study are selected based on *p*-value (either by ordering all *p*-values and selecting some number, or by choosing all with a *p*-value below some threshold), the observed case-control odds ratios at those variants are upwards-biased when used as estimates of the true odds-ratios of these variants between cases and controls in the population [10]. This bias is highest amongst variants for which the true log-odds-ratio is 0 (non-associated).

A standard replication procedure can be considered as enabling an unbiased effect size estimate [11]; for non-associated variants, this estimate has expectation 0. If controls are reused in the replication procedure, the estimate of effect size for associated variants from the replication procedure is no longer unbiased (since the original control samples are reused), and summary statistics from the replication procedure cannot be used directly as estimates of effect size (although estimates can still be made by considering summary statistics *p*_*d*_, *p*_*r*_ if these can be calculated). After sharing controls, the effect size estimate for null variants in the replication procedure is similarly biased, and the adjustment *β*→*β*^∗^/*β*^⊥^ corresponds to an adjustment for this effect.

### Differences in power between methods

The power difference between methods B and A was analysed systematically by considering the behaviour of GWAS data across a range of values of $(n_{0},n_{1},n_{0}^{\prime },n_{1}^{\prime })$. In each calculation, genetic data was considered for a single common SNP with average minor allele frequency across cases and controls equal to 0.1, with a given effect size between cases and controls quantified by log-odds ratio. Varying ratios $n_{1}/n_{1}^{\prime }$, $n_{0}^{\prime }/n_{1}^{\prime }$ were considered, with $n_{0} + n_{0}^{\prime } + n_{1} + n_{1}^{\prime }$ held constant at 20,000 samples (Fig. 1).

**Fig. 2:**
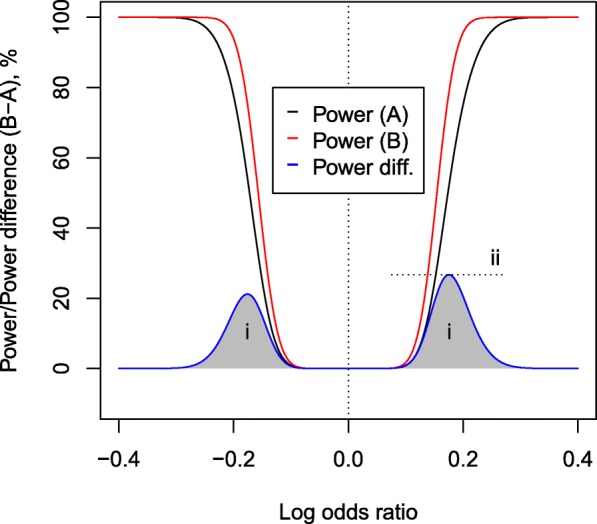
Power of both methods is equivocal at high effect sizes (high absolute log odds ratios) and at low effect sizes (log odds ratio near zero). The main region in which power can differ is at moderate effect sizes. A good metric for difference in power is the average difference in power (marked ‘i’). The maximum difference in power (marked ‘ii’) is also considered. This plot shows analytic rather than simulated results

At large effect sizes (in GWAS terms, large allelic differences between case and control cohorts) both methods have power approaching 1, so the difference is slight. Similarly, at very small effect sizes, both methods have power near zero. Since the only power differences are at moderate effect sizes, the main metric for power difference used in this paper was the average effect size difference (Fig. 2). Considering power of A and power of B as functions Power_*A*_(*x*), Power_*B*_(*x*) of an underlying log-odds ratio *x*, the average power difference was defined as

1 $$ \int_{-\infty}^{\infty} \left(\text{Power}_{B}(x) - \text{Power}_{A}(x) \right) dx  $$

The maximum power difference:

2 $$ \max_{x \in (-\infty,\infty)} \left(\text{Power}_{B}(x) - \text{Power}_{A}(x) \right)  $$

was also considered.

**Fig. 3:**
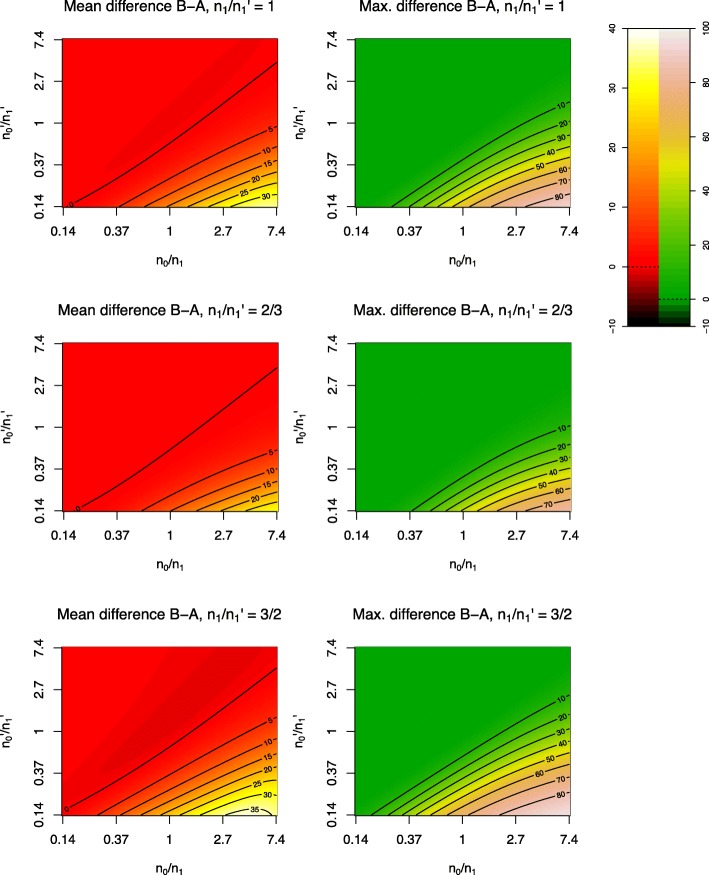
General power differences (%) between methods A and B. Mean power difference is taken as the integral of power difference between methods B and A (see above) over $\mathbb {R}$ with respect to log-odds ratio. In all cases, 20 000 samples are used overall for a SNP with MAF 0.1, with cutoffs *α*=5×10^−6^, *β*=5×10^−4^, *γ*=5×10^−8^

Figure 3 shows average power difference at various study sizes for typical *α*, *β*, *γ* values (*α*=5×10^−6^, *β*=5×10^−4^, *γ*=5×10^−8^). The difference is typically highest when the ratio of controls to cases is high in the discovery cohort and low or equal in the replication cohort, and the number of cases in the discovery cohort is larger than the number in the replication cohort. Power to detect SNPs in *H*_1_ is typically highest in method C, second-highest in method B, and lowest in method A.

### Recommended applications

To demonstrate areas where this approach is applicable, several examples are constructed or sourced from the GWAS field in which the procedure of sharing controls or cases will improve power or type-1 error profile of the two-stage testing procedure or enable some form of orthogonal replication to be performed.

#### Assumptions

In order to use method B or C, it must be assumed that cohort *C*_0_ and $C_{0}^{\prime }$ are sampled from similar enough populations to be comparable to *C*_1_ and $C_{1}^{\prime }$. A reasonable check on whether the method is appropriate is whether the cohorts *C*_0_ and $C_{0}^{\prime }$ could be interchanged without compromising matching between cases and controls in the discovery or validation studies (possibly with the inclusion of strata or covariates in the genetic risk model). An important caveat of methods B and C is sacrifice of control over errors arising from aberrance in *C*_0_ (method B) or $C_{0} \cup C_{0}^{\prime }$ (method C), so an assumption must be made that variables affected by confounding or measurement error in these cohorts are understood to be distinguishable from true associations by quality-control measures only. Variants which are aberrant in the same direction in both discovery and control cohorts - that is, $\text {sign}(\mu _{1}-\mu _{0})=\text {sign}(\mu _{1}^{\prime }-\mu _{0}^{\prime }) \neq 0$ - cannot be distinguished from true associations without the use of external data.

Post-hoc assessment of all putative hits should be performed to check for genotyping errors [12] and assess whether the hit could have arisen from aberrance in *C*_0_.

#### Conventional GWAS

**Fig. 4:**
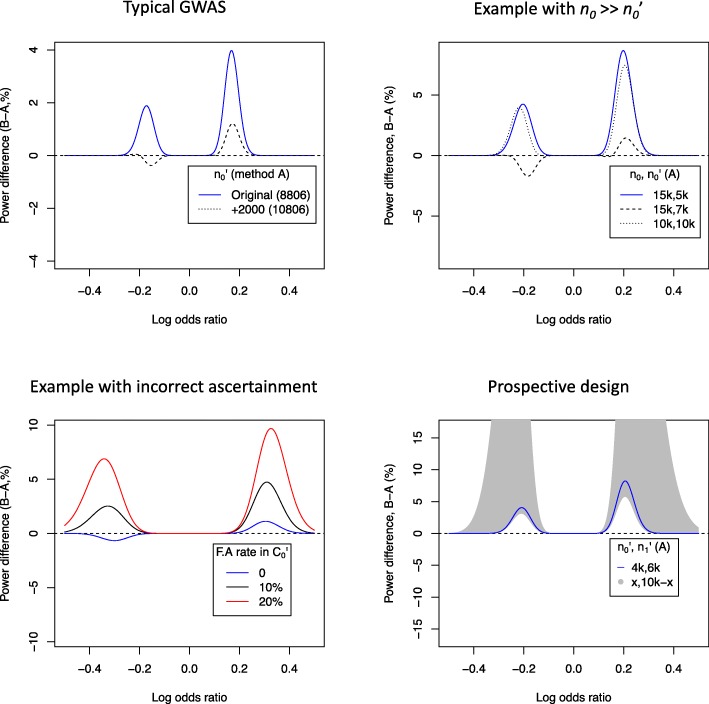
Examples of comparison of power of methods A and B. In all panels, a positive odds ratio corresponds to a deleterious mutation and average MAF is 10%. The top two panels show comparisons of method B with $n_{0}^{\prime }$ fixed against method A with varying $n_{0}^{\prime }$. The top left panel has $(n_{0},n_{1},n_{0}^{\prime },n_{1}^{\prime }) = (20169, 5539,8806,6768)$ (values from a GWAS on RA [13]), and the top right panel $(n_{0},n_{1},n_{0}^{\prime },n_{1}^{\prime }) = (15000, 5000,5000,5000)$. Both panels use (*α*,*β*,*γ*)=(5×10^−6^,5×10^−4^,5×10^−8^). The bottom left panel demonstrates the effect of false-ascertainment (F.A) in $C_{0}^{\prime }$; when cases are mis-ascertained as controls. In this case, (*α*,*β*,*γ*)=(1×10^−4^,1×10^−3^,5×10^−8^), reflecting values used in the paper [14]. The bottom right panel demonstrates a prospective scenario with 10000 samples for replication. Method B with (*n*_0_,*n*_1_) as above, $(n_{0}^{\prime },n_{1}^{\prime })=(4000,6000)$ is more powerful than any design using method A (grey region; $n_{0}^{\prime } \in (1000,9000)$; $n_{1}^{\prime }=10000-n_{0}^{\prime }$)

Method B is applicable in several cases in large conventional GWAS, particularly when then ratio of controls to cases in the discovery cohort is larger than that in the replication cohort. In a relatively recent GWAS on rheumatoid arthritis [13] with comparable sample populations for discovery and replication cohorts, method B could be used to attain greater power than method A for a fixed type-1 error rate. Assuming that summary statistics are well-approximated by binomial tests of allelic differences (so covariates and strata used in computation of summary statistics have only small effects), the improvement in power is around 4% for SNPs with an odds-ratio of 1.3, MAF 0.1, and is positive across all odds ratios. More than 2000 additional controls in $C_{0}^{\prime }$ would be needed to increase power by this amount (Fig. 4, top left).

Small power advantages such as this may make minimal difference in a single study, although since they require no extra cost, are worth attaining if possible. The power of method B is generally considerably higher than method A when *n*_0_>*n*_1_ and $n_{0}^{\prime } \approx n_{1}^{\prime }$. Power advantages may be more substantial in some cases; for example, a study with $(n_{0},n_{1},n_{0}^{\prime },n_{1}^{\prime })=(15 000,5000,5000,5000)$, method B enables a power increase of up to 8% (Fig. 4, top right panel). To achieve comparable performance with method A, around 2000 additional controls would be necessary in the replication cohort. Method B with $(n_{0},n_{0}^{\prime })=(15000,5000)$ is also more powerful than method A would be if controls were divided equally between *C*_0_ and $C_{0}^{\prime }$ (see Fig. 4, top right panel).

#### Difficult control ascertainment

An important application of the method presented in this paper is in studies for which ‘control’ samples are expensive or difficult to ascertain. This is often the case in comparative studies between disease subtypes. In such studies, sharing controls can improve power substantially, especially if a proportion of samples in the replication cohort are falsely assigned to the control cohort (see “Methods” section).

An international GWAS on fronto-temporal dementia in 2014 [14] is an example in which sharing controls may be beneficial. The study had sample sizes $(n_{0},n_{1},n_{0}^{\prime },n_{1}^{\prime })=(4308,2154,5094,1372)$. Control samples in the discovery phase were assessed for current neurological disease, and were used in previous studies on Parkinson’s disease, indicating a high degree of reliability. Control samples in the replication phase were collected from the same geographic distribution as cases, but were not explicitly used in previous neurological studies, suggesting better control ascertainment amongst the discovery cohort.

In this study, sharing controls could allow for a more strongly-ascertained control cohort, and reduce the effects of confounders affecting $C_{1}^{\prime }$ (see Fig. 4, bottom left panel). At typical values *α*=1×10^−4^, *β*=1×10^−3^, *γ*=5×10^−8^, power is nearly equivalent between the two methods assuming all controls are genuine. However, with 10% misascertainment in $C_{1}^{\prime }$, the power advantage of method B is up to 5%. Given the near-identical distribution of cases in the discovery and validation cohort, cases could alternatively be shared, leading to a power increase of up to 6%.

### Prospective study design

Studies may be planned and powered with the assumption that samples may be shared. For certain restrictions on sample numbers, this can provide the potential for greater power than would be attainable by restricting to an independent-controls design. For instance, if we seek to validate hits on a GWAS with 10,000 controls and 5000 cases, and can afford to genotype a further 10,000 samples, power is higher after recruiting 4000 additional controls and 6000 additional cases and sharing controls than can be achieved from any independent-control study design (Fig. 4, bottom right panel).

This may be a common scenario if controls are sourced from a known database rather than specifically recruited for the study.

#### Partial replication

In circumstances where case recruitment is difficult, as in studies of rare diseases, an assessment of replicability may be made by re-testing results from a discovery phase with a new control set only. This can enable the use of control cohorts which only partially match the case cohort.

In a GWAS on pemphigus vulgaris [15], a rare disease primarily affecting individuals of Ashkenazi Jewish ethnicity, the discovery cohorts were sampled from Jewish populations, with age- and population- matched controls. Control cohorts were small ($(n_{0},n_{1},n_{0}^{\prime },n_{1}^{\prime })=(100,400,59,285)$), potentially due to difficulty recruiting both ethnically- and geographically-matched controls.

Method C could be used in this instance to enable a larger control set and greater power. If a control cohort of Ashkenazi individuals could be assembled without requiring geographic matching with the case set, it would be inappropriate to use as a sole control cohort against the existing case cohort, due to the potential for geographic confounding. However, such a cohort could be used as either *C*_0_ or $C_{0}^{\prime }$ in method C, with the existing ethnically- and geographically- matched controls serving as the other cohort. In this way, the power advantage of the larger cohort could be used while maintaining control over potential aberrance in the larger control group.

Method C enables computation of power and type-1 error rates, and comparison to alternative designs with cases split into smaller independent discovery and validation cohorts (method A). Testing a case cohort against two separate control cohorts is almost always more powerful for a fixed type-1 error rate than splitting the case cohort in two and performing method A (see Additional file 1: Figures S1 and S2).

### Choice of thresholds

The designation of explicit thresholds *α*, *β*, *γ* in a two-stage study may not appear to reflect many real-life designs, but in general most studies will use it in some form, even if the thresholds are not directly stated. Heuristically, *α* is used as an initial ‘triage’ step, to reduce data dimensionality, *β* (which is usually less stringent than *α* to allow for some regression to the mean in true associations) is used as a check, and *γ* is used as a definitive test for association amongst candidate variants.

Because studies are usually limited by cost or resources, a given number of variants are selected to pass through to the replication step, rather than following up all variants passing a predetermined threshold, which complicates assessment of summary statistics [11]. However, in practice, researchers will have an implicit or explicit maximum allowable *p*-value for a variant to proceed to replication. If, for example, resources were available to follow-up 100 variants, but the 100th smallest Bonferroni-corrected *p*_*d*_ value was >1, the variant would not generally be followed up. It is this implicit threshold - representing the maximum allowable *p*_*d*_ value which would be be deemed acceptable - which is considered to be *α*. A similar implicit threshold at the replication stage is the effective value of *β*. If no thresholds *α*, *β* are used (that is, *α*=*β*=1), then the procedure can be considered as a standard meta-analysis of the discovery and replication studies, and cannot be improved upon by combining controls at the replication stage.

If the method proposed in this paper is to be considered in a study, the values *α*, *β*, *γ* should be determined by the values which would otherwise have been used in a standard replication procedure. In the context of GWAS analysis, the threshold *γ*=5×10^−8^ should be retained, and the values *α*, *β* should reflect the implicit maximum allowable level above. The corresponding *β*^∗^/*β*^⊥^ values can then be determined. As in any statistical procedure, the overall false-positive rate should be considered along with the cost of following up false-positives.

## Discussion

This paper proposes a method to improve efficiency of data use in a replication procedure, adding to the body of methods for comparison of high-dimensional case-control studies. For many common study sizes, the method can reduce the cost of replication, or increase power of discovery. The adapted method is simple to apply, only requiring modification of a single association threshold.

A standard replication procedure (or more general comparison of case-control studies) with independent control datasets does not make use of the information that the unconditional expected values of variables in control datasets are, in principle, the same. Conditional on *p*_*d*_≤*α*, *m*_0_ is biased away from *m*_1_ (since the effect size is biased upwards), and this bias is greatest for non-associated variants. If the observed difference *m*_1_−*m*_0_ is large even accounting for this bias, and the observed difference $m_{1}^{\prime } - m_{0}^{\prime }$ is small but consistent in direction with *m*_1_−*m*_0_, we intuitively expect that the variant is disease associated, with the observed *m*_1_−*m*_0_ value being larger than its unconditional expectation, and the $m_{1}^{\prime }- m_{0}^{\prime }$ value being smaller. In a standard replication procedure, the variant would be declared null on the basis of $m_{1}^{\prime }-m_{0}^{\prime }$ being small, but in the shared controls procedure, some information from the first study is allowed to propagate through to the second. A meta-analysis in which observed values of both *m*_1_ and *m*_0_ are allowed to propagate information is stronger still, but this cannot in itself detect aberrance in $C_{1}^{\prime }$.

Correspondingly, a more stringent threshold *β*^∗^/*β*^⊥^ is needed to account for the bias in $m_{1}^{\prime }$ conditioning on *p*_*d*_<*α*, and the differential in power between the standard replication procedure and the two proposed here relates to the trade-off between these two effects. By considering which method has the highest power in a given circumstance, the same dataset can in theory yield more information when controls are shared, while retaining some of the systematic error-detecting properties of the standard replication procedure.

The most important caveat of these methods is the loss of systematic type-1 error rate control for null SNPs which are aberrant in *C*_0_. Control of such errors must not be sacrificed entirely, but in some circumstances it may be satisfactory to assess such errors on a SNP-by-SNP basis. Such assessment is important and standard for all proposed GWAS hits under any method [16] in the interests of quality control. In method C, control over aberrance in $C_{0}^{\prime }$ is additionally lost; however, since this method is largely applicable when $C_{0} \cup C_{0}^{\prime }$ is a single homogeneous control (or case) cohort, there is no way that aberrance in the cohort can be systematically identified by comparison with other cohorts.

Somewhat better control of the type-1 error rate can often be achieved for SNPs with aberrance in *C*_1_ or $C_{0}^{\prime }$. This may incentivise the use of this method when confidence in the representativeness of these cohorts is low compared to that of *C*_0_. The type 1 error rate is somewhat increased for SNPs with aberrance in $C_{1}^{\prime }$, although as it remains bounded by *α*, this increase is not a major problem.

The two-stage validation procedure is similar to a meta-analysis of the discovery and validation experiments, for which several adaptations to shared-control designs have been proposed [3, 4]. However, there are several important distinctions which necessitate an alternative approach in this case. Firstly, not all variables are measured in the second (replication) study; we are restricted to analysis of variables reaching a given observed effect size. Secondly, the studies to be ‘meta-analysed’ are not complete, in the sense that there may be residual confounding; a strong effect size in the meta-analysis alone is not adequate evidence for association and some level of association (with consistent direction) is additionally required in both constituent studies.

The method is inapplicable when replication is performed on cohorts from completely distinct geographic groups, although there can be some difference in geographic distribution between control sets if this is controlled for in computing summary statistics. The method is most applicable when control groups are sampled from similar populations and genotyped on similar platforms. The method proposed in this paper is not universally applicable, and may only yield a modest increase in power, at the cost of changing sensitivity to different types of errors. However, it is in the interest of all researchers to use data as efficiently as possible, and methods such as this which may provide improvements without additional cost in resources should be considered as analytical options.

The widespread discoveries of the GWAS field have led to corresponding increases in complexity of phenotypic definitions, with ever-finer delineations of disease types of ever-rarer prevalence. The genetic analysis of such complex phenotypes is necessarily comparative; there is little use understanding the genetics of a rare disease subtype except in the context of the genetics of the disease in general. Such analyses necessitate GWAS and other comparative studies between rare phenotypic types [17], with ‘controls’ meaning the better-characterised disease subphenotype in this sense, as well as between cases and controls. Rare disease subtypes are often afflicted with ascertainment difficulties, leading to varying degrees of expected aberrance in disease cohorts. Within this paradigm, the applicability of this method is likely to expand.

## Conclusions

This paper details a method in which controls are shared in the replication phase of a two-stage association study. Sharing controls can improve the power of the two-stage procedure at a fixed type-1 error rate. The action of sharing controls changes the spectrum of sensitivity to systematic errors caused by confounders affecting one of the study cohorts, and this should be accounted for if the shared-control design is used. Adaptations of the method can enable a partial replication to be performed with only a new control cohort, or to enable robustness to mis-ascertainment of control samples in the replication cohort.

## Methods

### Definitions

Denote *z*_*d*_, *z*_*r*_, *z*_*s*_, *z*_*m*_, *z*_*c*_ as the signed z-scores corresponding to *p*_*d*_, *p*_*r*_, *p*_*s*_, *p*_*m*_ and *p*_*c*_ respectively (where subscripts *d*, *r*, *s*, *m*, *c* are as defined in the “Results” section), so *z*_*d*_=±*Φ*^−1^(*p*_*d*_/2) and so on (where *Φ*, *Φ*^−1^ are the standard normal CDF and quantile functions). Define $\phantom {\dot {i}\!}z_{\alpha }, z_{\beta }, z_{\beta ^{*}}, z_{\beta ^{\perp }}, z_{\gamma }$ as the positive corresponding thresholds for *α*, *β*, *β*^∗^, *β*^⊥^, *γ* respectively, so *z*_*α*_=−*Φ*^−1^(*x*/2) etc. Other than (*z*_*d*_,*z*_*r*_), all pairs of z-scores are correlated under $H_{0}^{=}$, with correlation estimable from sample sizes or empirically if covariates are used (Additional file 1: Appendix 1). Denote *ρ*_*ij*_ as the correlation between *z*_*i*_ and *z*_*j*_, (*i*,*j*)∈{*d*,*r*,*s*,*m*,*c*}^2^, and set

3 $$  \begin{aligned} \Sigma_{A} = \text{var} \left((z_{d} \, z_{r} \, z_{m})^{t} \right) \\ \Sigma_{B} = \text{var} \left((z_{d} \, z_{s} \, z_{m})^{t} \right)\\ \Sigma_{C} = \text{var} \left((z_{c} \, z_{s} \, z_{m})^{t} \right)\end{aligned}  $$

For *i*∈{*d*,*r*,*s*,*m*,*c*} define *ζ*_*i*_=*E*(*z*_*i*_), where the expectation is conditional on the SNP in question. For SNPs in $H_{0}^{=}$, *ζ*_*i*_≡0 for all *i*, but this may not hold for SNPs in $H_{0}^{\cup } \setminus H_{0}^{=}$. In theoretical working, aberrance in groups is characterised by values *ζ* rather than log-odds ratios. Define *R*_*A*_, *R*_*B*_, *R*_*C*_ as the false-positive rates for a SNP of interest in methods A, B and C respectively.

### General type 1 error rate

**Fig. 5:**
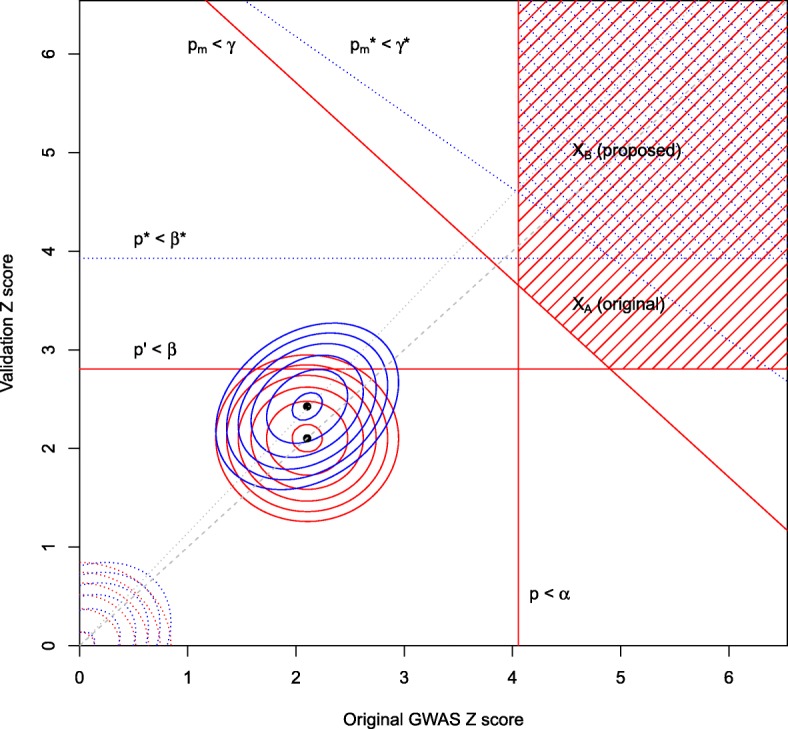
Replication with shared controls. Red and blue shaded areas are regions where a pair of observed *Z* scores are deemed a ‘hit’ in the (+,+) quadrant under method A/B respectively. The value *z*_*m*_ is almost linearly dependent on (*z*_*d*_,*z*_*r*_) and on (*z*_*d*_,*z*_*s*_) (Additional file 1: Appendix 1). Solid red/blue ellipses indicate contours of the distribution of observed *Z* scores for a typical non-null SNP under methods A and B, and dashed ellipses indicate contours for a null SNP

The values *β*^∗^, *β*^⊥^ are chosen to satisfy

4 $$ {{}\begin{aligned} 2\int_{z_{\alpha}}^{\infty}\! \int_{z_{\beta^{*}}}^{\infty}\! \int_{z_{\gamma}}^{\infty}\! N_{\Sigma_{B}}\!\! \left(\!\begin{array}{c} z_{d}\\ z_{s}\\ z_{m} \end{array}\!\right)\! dz_{m} dz_{s} dz_{d} & \,=\, 2\int_{z_{\alpha}}^{\infty}\! \int_{z_{\beta^{\perp}}}^{\infty}\! \int_{z_{\gamma}}^{\infty}\! N_{\Sigma_{C}}\!\! \left(\!\begin{array}{c} z_{c}\\ z_{s}\\ z_{m} \end{array}\!\right) dz_{m} dz_{s} dz_{c} \\ &= 2\int_{z_{\alpha}}^{\infty}\! \int_{z_{\beta}}^{\infty}\! \int_{z_{\gamma}}^{\infty}\! N_{\Sigma_{A}}\!\! \left(\!\begin{array}{c} z_{d}\\ z_{r} \\ z_{m} \end{array}\!\right)\! dz_{m} dz_{r} dz_{d}\\ &= \Pr\left(p_{d}<\alpha,p_{r}<\beta,p_{m}<\gamma|H_{0}^{=}\right)  \end{aligned}}  $$

thus conserving the type 1 error rate (denoted *P*_0_) against $H_{0}^{=}$ between methods (Fig. 5). If no threshold is used on *p*_*m*_ (ie, *γ*=1), then *β*^∗^, *β*^⊥^ satisfy

5 $$\begin{array}{*{20}l} \Pr(p_{d}<\alpha,p_{s}<\beta^{*}|H_{0}^{=}) &= \Pr\left(p_{c}<\alpha,p_{s}<\beta^{\perp}|H_{0}^{=}\right)  \\ &=\Pr\left(p_{d}<\alpha,p_{r}<\beta|H_{0}^{=}\right)  \\ &= \alpha \beta  \end{array} $$

since $z_{d} \perp \perp z_{r}|H_{0}^{=}$. Definition Eq. (4) will be considered a generalisation of definition Eq. (5), with results established first for *β*^∗^ as per definition Eq. (5) and extending where possible to definition Eq. (4).

For *β*^∗^ defined as per definition Eq. (5) we have (see Additional file 1: Appendix 2)

6 $$ {\begin{aligned} &{\lim}_{z_{\alpha} \to \infty} \frac{z_{\beta^{*}}}{\sqrt{1-\rho_{ds}^{2}}z_{\beta} + \rho_{ds} z_{\alpha}} &= 1 \\ & {\lim}_{z_{\alpha} \to \infty} \frac{z_{\beta^{\perp}}}{\sqrt{1-\rho_{cs}^{2}}z_{\beta} + \rho_{cs} z_{\alpha}} &= 1 \end{aligned}}  $$

approaching from above, so $z_{\beta ^{*}} > \max \left (z_{\beta }, \sqrt {1-\rho _{ds}^{2}}z_{\beta }\right. \left. + \rho _{ds} z_{\alpha }{\vphantom {\sqrt {1-\rho _{ds}^{2}}z_{\beta }}} \right)$ and $z_{\beta ^{\perp }} > \max \left (z_{\beta },\sqrt {1-\rho _{cs}^{2}}z_{\beta } + \rho _{cs} z_{\alpha } \right)$. As defined by Eq. 5, $\phantom {\dot {i}\!}z_{\beta ^{*}}, z_{\beta ^{\perp }}$ are also asymptotically linear in *z*_*α*_, *z*_*γ*_, *z*_*β*_ as the former two tend to *∞*, with some constraints (Additional file 1: Appendix 2.1), although the limit does not necessarily approach from above. For both definitions, *β*^⊥^<*β*^∗^<*β* (Additional file 1: Appendix 2.2).

### Empirical computations

Define *N*_*Σ*_(**z**) as the *pdf* of the multivariate normal with mean 0 and variance *Σ* at **z**. Determination of covariance is described in Additional file 1: Appendix 1. Given *ζ*_*d*_, *ζ*_*r*_, *ζ*_*s*_, *ζ*_*m*_, the probability of rejecting the null for a given SNP using method A is

7 $$\begin{array}{*{20}l} &\int_{z_{\alpha}-\zeta_{d}}^{\infty} \int_{z_{\beta} - \zeta_{r}}^{\infty} \int_{z_{\gamma} - \zeta_{m}}^{\infty} N_{\Sigma_{A}} \left((z_{d} \, z_{r} \, z_{m})^{t}\right) dz_{m} dz_{r} dz_{d}  \\ &+ \int_{z_{\alpha}+\zeta_{d}}^{\infty} \int_{z_{\beta} + \zeta_{r}}^{\infty} \int_{z_{\gamma} + \zeta_{m}}^{\infty} N_{\Sigma_{A}} \left((z_{d} \, z_{r} \, z_{m})^{t}\right) dz_{m} dz_{r} dz_{d} \end{array} $$

and using method B

8 $$\begin{array}{*{20}l} &\int_{z_{\alpha}-\zeta_{d}}^{\infty} \int_{z_{\beta} - \zeta_{s}}^{\infty} \int_{z_{\gamma} - \zeta_{m}}^{\infty} N_{\Sigma_{B}} \left((z_{d} \, z_{s} \, z_{m})^{t}\right) dz_{m} dz_{s} dz_{d}  \\ &+ \int_{z_{\alpha}+\zeta_{d}}^{\infty} \int_{z_{\beta} + \zeta_{s}}^{\infty} \int_{z_{\gamma} + \zeta_{m}}^{\infty} N_{\Sigma_{B}} \left((z_{d} \, z_{s} \, z_{m})^{t}\right) dz_{m} dz_{s} dz_{d} \end{array} $$

If $\frac {n_{0}}{n_{1}}=\frac {n_{0}^{\prime }}{n_{1}^{\prime }}$, matrix *Σ*_*A*_ is singular (Additional file 1: Appendix 1), in which case *z*_*m*_=*ρ*_*dm*_*z*_*d*_+*ρ*_*vm*_*z*_*v*_ and the expression above may be reduced to a two-dimensional integral over a more complex region (Fig. 5). Matrix *Σ*_*C*_ is generally singular, so the formula $z_{m} = \frac {\rho _{cs}\rho _{sm}-\rho _{cm}}{\rho _{cs}^{2} - 1} z_{d} + \frac {\rho _{cs}\rho _{cm} - \rho _{sm}}{\rho _{cs}^{2} - 1} z_{s}$ is used to reduce the integral in a similar way. A similar formula may be used if *Σ*_*B*_ is nearly singular.

In Fig. 3, mean power difference is determined as the integral of the power difference with respect to the log-odds ratio over the real line, as discussed in the “Results” section.

### Study sizes, odds ratios and allele frequencies

Consider a study with *n*_0_ controls and *n*_1_ cases, with underlying allele frequencies *μ*_0_ and *μ*_1_ in cases and observed allele frequencies *m*_0_, *m*_1_. Let *Z* be a signed Z-score derived from a GWAS *p*-value against the null hypothesis *μ*_0_=*μ*_1_. Considering *Z* to be proportional to the log-odds-ratio divided by its standard error, we have:

9 $$\begin{array}{*{20}l} E(Z) &\approx E\left(\frac{\log\left(\frac{m_{1}(1-m_{0})}{m_{0}(1-m_{1})}\right)}{SE\left(\log\left(\frac{m_{1}(1-m_{0})}{m_{0}(1-m_{1})}\right)\right)}\right)  \\ &\approx E\left(\frac{\log\left(\frac{m_{1}}{1-m_{1}}\right) - \log\left(\frac{m_{0}}{1-m_{0}}\right)}{\sqrt{\frac{2}{m_{1} (1-m_{1}) n_{1}} + \frac{2}{m_{0} (1-m_{0}) n_{0}} }}\right) \end{array} $$

Setting *δ*=*m*_1_−*m*_0_, $\bar {m} = \frac {n_{0} m_{0} + n_{1} m_{1}}{n_{0}+ n_{1}} m_{0}=\bar {m} - k \delta $, $m_{1}=\bar {m} + k \delta $ for some *k*, we have

10 $$ {{}\begin{aligned} \log\left(\frac{m_{1}}{1-m_{1}}\right) - \log\left(\frac{m_{0}}{1-m_{0}}\right) &= \frac{\delta}{\bar{m}(1-\bar{m})} + O\left(\delta^{2}\right) \\ \sqrt{\frac{2}{m_{1} (1-m_{1}) n_{1}} + \frac{2}{m_{0} (1-m_{0}) n_{0}}} &= \sqrt{\frac{2 (n_{0}+ n_{1})}{\bar{m}(1-\bar{m})n_{0} n_{1}}} + O(\delta) \end{aligned}}  $$

so

11 $$\begin{array}{*{20}l} E(Z) &\approx \sqrt{\frac{2 n_{0} n_{1}}{n_{0}+n_{1}}} E\left(\frac{\delta}{\sqrt{\bar{m}(1-\bar{m})}} + O(\delta^{2}) \right)  \\ &\approx \sqrt{\frac{2 n_{0} n_{1}}{n_{0}+n_{1}}} \frac{\mu_{1}-\mu_{0}}{\sqrt{\bar{\mu}(1-\bar{\mu})}} \end{array} $$

where $\bar {\mu } = \frac {n_{0}\mu _{0}+n_{1} \mu _{1}}{n_{0}+n_{1}}$. Hence

12 $$ {{}\begin{aligned} \zeta_{d} &= \sqrt{\frac{2 n_{0}n_{1}}{n_{0}+n_{1}}}\frac{\mu_{1}-\mu_{0}}{\sqrt{\bar{\mu}(1-\bar{\mu})}} \zeta_{r} = \sqrt{\frac{2 n_{0}^{\prime}n_{1}^{\prime}}{n_{0}^{\prime}+n_{1}^{\prime}}}\frac{\mu_{1}^{\prime}-\mu_{0}^{\prime}}{\sqrt{\bar{\mu}(1-\bar{\mu})}} \\ \zeta_{s} \!&=\! \sqrt{\frac{2 (n_{0}+n_{0}^{\prime})n_{1}^{\prime}}{n_{0}+n_{0}^{\prime}+n_{1}^{\prime}}}\frac{\mu_{1}^{\prime}-\frac{\mu_{0}n_{0}+\mu_{0}^{\prime}n_{0}^{\prime}}{n_{0}+n_{0}^{\prime}}}{\sqrt{\bar{\mu}(1-\bar{\mu})}} \zeta_{c} = \sqrt{\frac{2 (n_{0}+n_{0}^{\prime})n_{1}}{n_{0}+n_{0}^{\prime}+n_{1}}}\frac{\mu_{1}-\frac{\mu_{0}n_{0}+\mu_{0}^{\prime}n_{0}^{\prime}}{n_{0}+n_{0}^{\prime}}} {\sqrt{\bar{\mu}(1-\bar{\mu})}}\\ \zeta_{m} &= \sqrt{\frac{2 (n_{0}+n_{0}^{\prime})(n_{1}+n_{1}^{\prime})}{n_{0}+n_{0}^{\prime}+n_{1}+n_{1}^{\prime}}}\frac{\frac{\mu_{1}n_{1}+\mu_{1}^{\prime}n_{1}^{\prime}}{n_{1}+n_{1}^{\prime}}-\frac{\mu_{0}n_{0}+\mu_{0}^{\prime}n_{0}^{\prime}}{n_{0}+n_{0}^{\prime}}}{\sqrt{\bar{\mu}(1-\bar{\mu})}}  \end{aligned}}  $$

where $\bar {\mu }$ varies between definitions (though it is taken to be approximately equal).

#### Estimation of covariance between Z scores

Correlation between Z-scores under *H*_0_ can be computed analytically with the following formulas (with *ρ*_*dr*_=0):

13 $$ {{} \begin{aligned} \rho_{dm} &=\frac{\sqrt{n_{0} n_{1}} \left(n_{0}+n_{0}^{\prime} + n_{1} + n_{1}^{\prime}\right)}{\sqrt{\left(n_{0}+n_{0}^{\prime}\right)\left(n_{1}+n_{1}^{\prime}\right)\left(2n_{0}+n_{0}^{\prime}\right)\left(2n_{1}+n_{1}^{\prime}\right)}} \\ \rho_{rm}&=\frac{\sqrt{n_{0}^{\prime} n_{1}^{\prime}} \left(n_{0}+n_{0}^{\prime} + n_{1} + n_{1}^{\prime}\right)}{\sqrt{\left(n_{0}+n_{0}^{\prime}\right)\left(n_{1}+n_{1}^{\prime}\right)\left(n_{0}^{\prime}+2n_{0}^{\prime}\right)\left(n_{1}+2n_{1}^{\prime}\right)}} \\ \rho_{ds} &= \sqrt{\frac{n_{0} n_{1} n_{1}^{\prime}}{\left(n_{0} + n_{0}^{\prime}\right)\left(n_{0}+n_{1}\right)\left(n_{0}+n_{0}^{\prime}+n_{1}^{\prime}\right)}} \\ \rho_{sm} &= \frac{\sqrt{n_{1}^{\prime}}\left(n_{0}+n_{0}^{\prime}+n_{1}+n_{1}^{\prime}\right)}{\sqrt{2\left(n_{0}+n_{0}^{\prime}\right)\left(n_{1}+n_{1}^{\prime}\right)\left(n_{1}+2n_{1}^{\prime}\right)}} \\ \rho_{cs} &= \frac{\sqrt{n_{1} n_{1}^{\prime}}\left(n_{0}+n_{0}^{\prime}\right)}{\sqrt{\left(2 n_{0}+n_{0}^{\prime}\right)\left(2 n_{1}+n_{1}^{\prime}\right)}} \\ \rho_{cm} &= \frac{\sqrt{n_{1}}\left(n_{0}+n_{0}^{\prime}+n_{1}+n_{1}^{\prime}\right)}{\sqrt{2\left(n_{0}+n_{0}^{\prime}\right)\left(n_{1}+n_{1}^{\prime}\right)\left(2n_{1}+n_{1}^{\prime}\right)}}  \end{aligned}}  $$

More general formulae are given in Additional file 1: Appendix 1.

#### Empirical estimation of covariance and *ζ* values

The above formulae allow *ζ* and *ρ* to be estimated in empirical computations. The estimates may be poor if covariates or strata are used in the computation of *z*_*i*_. Correlation may be estimated in several ways:

1. If strata alone are used, or covariates are adjusted for in an analogous way to strata, correlations *ρ*_*ij*_ between z-scores is estimable using analytic formulas (see Additional file 1: Appendix 1).
2. If a set of variants known to be in $H_{0}^{=}$ is available, the sample correlation between observed z-scores at these variants can be used as an estimator for values *ρ*_*ij*_,
3. A set of genotypes can be simulated for each sample for a set of variants in $H_{0}^{=}$. Z-scores corresponding to these variants can then be computed under the same correlation structure, and the sample correlation between these Z-scores.

Estimates of values *ζ* corresponding to given log-odds ratios and minor allele frequencies can be estimated in a similar way to method 1; that is, by simulating variants with given underlying odds-ratios between cases and controls, computing *z* scores using the same method and covariance structure as used in the main study, and setting the relevant value of *ζ* to the observed mean *z* score.

#### False ascertainment

In general, for a true association, $\mu _{0}=\mu _{0}^{\prime }$ and $\mu _{1}=\mu _{1}^{\prime }$. If some proportion *κ* of samples in $C_{0}^{\prime }$ are incorrectly assigned and come from the case population, then $\mu _{0}^{\prime } = (1-\kappa)\mu _{0} + \kappa \mu _{1}$. This lowers the absolute values of *ζ*_*r*_, *ζ*_*s*_ and *ζ*_*m*_, reducing the probability that the *z*_*r*_ score for the SNP will reach the requisite threshold *β* and hence reducing the power to detect the SNP using method A. This loss of power is lowered when using methods B or C.

### Type 1 error rates

#### Aberrance in *C*_1_

For SNPs aberrant in only *C*_1_ we have *ζ*_*d*_≠0, *ζ*_*c*_≠0, *ζ*_*m*_≠0, and *ζ*_*r*_=*ζ*_*s*_=0.

*R*_*A*_, *R*_*B*_, *R*_*C*_ can be considered as functions of *ζ*_*d*_. As *ζ*_*d*_→0, *R*_*A*_,*R*_*B*_,*R*_*C*_→*P*_0_ (Eq. 4). As *ζ*_*d*_→±*∞*, $R_{A} \to \frac {\beta }{2}$, $R_{B} = \frac {\beta ^{*}}{2}$ and $R_{C} = \frac {\beta ^{\perp }}{2}$. For positive *ζ*_*d*_ both *R*_*A*_ and *R*_*B*_ are increasing (and both are symmetric in *ζ*_*d*_) so $R_{A}<\frac {\beta }{2}$, $R_{B} < \frac {\beta ^{*}}{2}$, $R_{C} < \frac {\beta ^{\perp }}{2}$ for all *ζ*_*d*_.

Since *β*^⊥^<*β*^∗^<*β* (often substantially), methods B and C are generally better at rejecting $H_{0}^{=}$ for such SNPs. In the simplified case where *z*_*γ*_=1, *R*_*A*_≥*R*_*B*_ universally (Additional file 1: Appendix 3.1). This typically holds for all *z*_*γ*_, except for small deviations in pathological cases.

In general, we consider aberrance which is only still present after any strata or covariates have been accounted for in the computation of *z* scores. If strata or covariates remove the effective aberrance between groups, the type-1 error rate is equivalent to that under $H_{0}^{=}$.

#### Aberrance in $C_{1}^{\prime }$

For SNPs aberrant in $C_{1}^{\prime }$, we have *ζ*_*d*_=0, *ζ*_*c*_=0, *ζ*_*r*_≠0, *ζ*_*s*_≠0 and *ζ*_*m*_≠0.

Again, *R*_*A*_,*R*_*B*_,*R*_*C*_→*P*_0_ as *ζ*_*r*_→0. As *ζ*_*r*_→±*∞*, $R_{A},R_{B},R_{C} \to \frac {\alpha }{2}$, and both are bounded by $\frac {\alpha }{2}$. Although *R*_*B*_ and *R*_*C*_ are typically higher than *R*_*A*_ in this case, since both have the same (typically conservative) upper bound, this is not typically a large sacrifice in type 1 error.

In the simplified case where *γ*=1, an approximate upper bound on *R*_*B*_−*R*_*A*_ is given by (Additional file 1: Appendix 4)

14 $$ \frac{\alpha}{2 \sqrt{2 \pi}} \left(\frac{k}{\sqrt{1-\rho^{2}}} - 1 \right) z_{\beta} \ll \frac{\alpha}{2}   $$

where

15 $$ k =\frac{\zeta_{s}}{\zeta_{r}} \approx \sqrt{\frac{(n_{0}+n_{0}^{\prime})(n_{0}^{\prime}+n_{1}^{\prime})}{n_{0}^{\prime}(n_{0}+n_{0}^{\prime}+n_{1}^{\prime})}}  $$

In practice, there is typically a similarly small difference between *R*_*C*_, *R*_*B*_ and *R*_*A*_ in the general case.

#### Aberrance in $C_{0}^{\prime }$

For SNPs aberrant in $C_{0}^{\prime }$, *ζ*_*d*_=0, *ζ*_*r*_≠0, *ζ*_*c*_≠0, *ζ*_*s*_≠0 and *ζ*_*m*_≠0. As for SNPs with aberrance in $C_{1}^{\prime }$, *R*_*A*_,*R*_*B*_,*R*_*C*_→*P*_0_ as *ζ*_*r*_→0 and as *ζ*_*r*_→±*∞*, $R_{A},R_{B} \to \frac {\alpha }{2}$, both bounded above by $\frac {\alpha }{2}$. *R*_*C*_, however, tends to 1 as *ζ*_*d*_→*∞*.

In method B the cohort *C*_0_ has a correcting effect on the replication study, meaning |*ζ*_*s*_|<|*ζ*_*r*_| and *R*_*B*_<*R*_*A*_.

For the simplified case where *γ*=1, a similar bound to 14 holds for the difference *R*_*A*_−*R*_*B*_ (note signs are reversed) with

16 $$ k^{\prime}=\frac{\zeta_{s}}{\zeta_{r}} \approx \sqrt{\frac{n_{0}^{\prime}(n_{0}^{\prime} + n_{1}^{\prime})}{(n_{0}+n_{0}^{\prime})(n_{0}+n_{0}^{\prime}+n_{1}^{\prime})}}  $$

in the place of *k*. The improvement in type-1 error rate for a SNP with aberrance in $C_{0}^{\prime }$ is generally larger than the loss with the same aberrance in $C_{1}^{\prime }$ (see methods), meaning that if aberrances are of similar prevalence and size in $C_{1}^{\prime }$ and $C_{0}^{\prime }$, method B will typically have a lower type-1 error rate than method A.

#### Aberrance in *C*_0_

Aberrance in *C*_0_ represents a serious problem in case-control study comparison. False-positive rates are generally worse under method B, and tend to 1 as *E*(*z*)→*∞*. If aberrances of this type are expected to be very frequent, this may preclude use of methods B or C.

However, aberrances of this type may be best detected retrospectively by examining aberrances between control groups at SNPs declared ‘hits’. This procedure is already a necessary quality-control procedure in method A [12, 16], as method A does not provide any control over differences between *C*_0_ and $C_{0}^{\prime }$. The number of SNPs reaching significance in the two-stage procedure is usually small enough that this examination is readily tractable.

#### Aberrance in two or more cohorts

If SNPs are aberrant in both *C*_1_ and $C_{1}^{\prime }$, or in both *C*_0_ and $C_{0}^{\prime }$, the effect on *R*_*A*_ and *R*_*B*_ is similar. If both cohorts are aberrant in the same direction, there is no way to differentiate the SNP from a genuine association on the basis of the genotype data alone. If cohorts are aberrant in different directions, then in both methods, the type-1 error rate is lower than for a null SNP with no aberration or aberration in only one cohort, as effect sizes for the discovery and replication cohorts are biased in opposite directions. The same typically holds if $C_{0}^{\prime }$ and *C*_1_, or *C*_0_ and $C_{1}^{\prime }$, are biased in the same direction.

If $C_{0}^{\prime }$ and $C_{1}^{\prime }$ or *C*_0_ and *C*_1_ are both biased in the same direction, *R*_*A*_ is generally lower than *R*_*B*_, as *ζ*_*s*_≠0. Both *R*_*A*_ and *R*_*B*_ are bounded by $\frac {\alpha }{2}$ in this case. In addition, a systematic bias in both replication groups (or both discovery groups) is likely to be due to a known confounder, the effect of which can be removed by performing a stratified test (as is typically good practice when confounders are known). Aberrance in opposite directions leads to *R*_*B*_>*R*_*A*_ in the first case, and a scenario similar to aberrance in *C*_0_ in the second case.

Aberrance in three or more cohorts corresponds to a chaotic scenario in which neither methods A,B, or C will reliably provide FPR control. Aberrance of this extent is typically detectable and removable using quality control procedures.

## Additional file

Additional file 1 Supplementary figures and appendices. Supplementary figures showing additional power comparisons, and appendices pertaining to the method. (PDF 941 kb)

## Abbreviations

GWAS: Genome-wide association study
MAF: Minor allele frequency
SNP: Single-nucleotide polymorphism
