# Supplementary material for: Combining controls can improve power in two-stage association studies
Source: BMC Genet. 2018 Oct 3;19:89. doi: 10.1186/s12863-018-0675-y (PMC6171163; doi:10.1186/s12863-018-0675-y)
Supplement: Supplementary file 1 — Supplementary figures and appendices. Supplementary figures showing additional power comparisons, and appendices pertaining to the method. (PDF 941 kb) [file 12863_2018_675_MOESM1_ESM.pdf]

Combining controls can improve power in two-stage association studies

Supplementary figures and appendices

James Liley[1]

[1]Department of Medicine, University of Cambridge, Addenbrooke's Hospital, Cambridge, CB2 0SP, UK. E: ajl88@cam.ac.uk

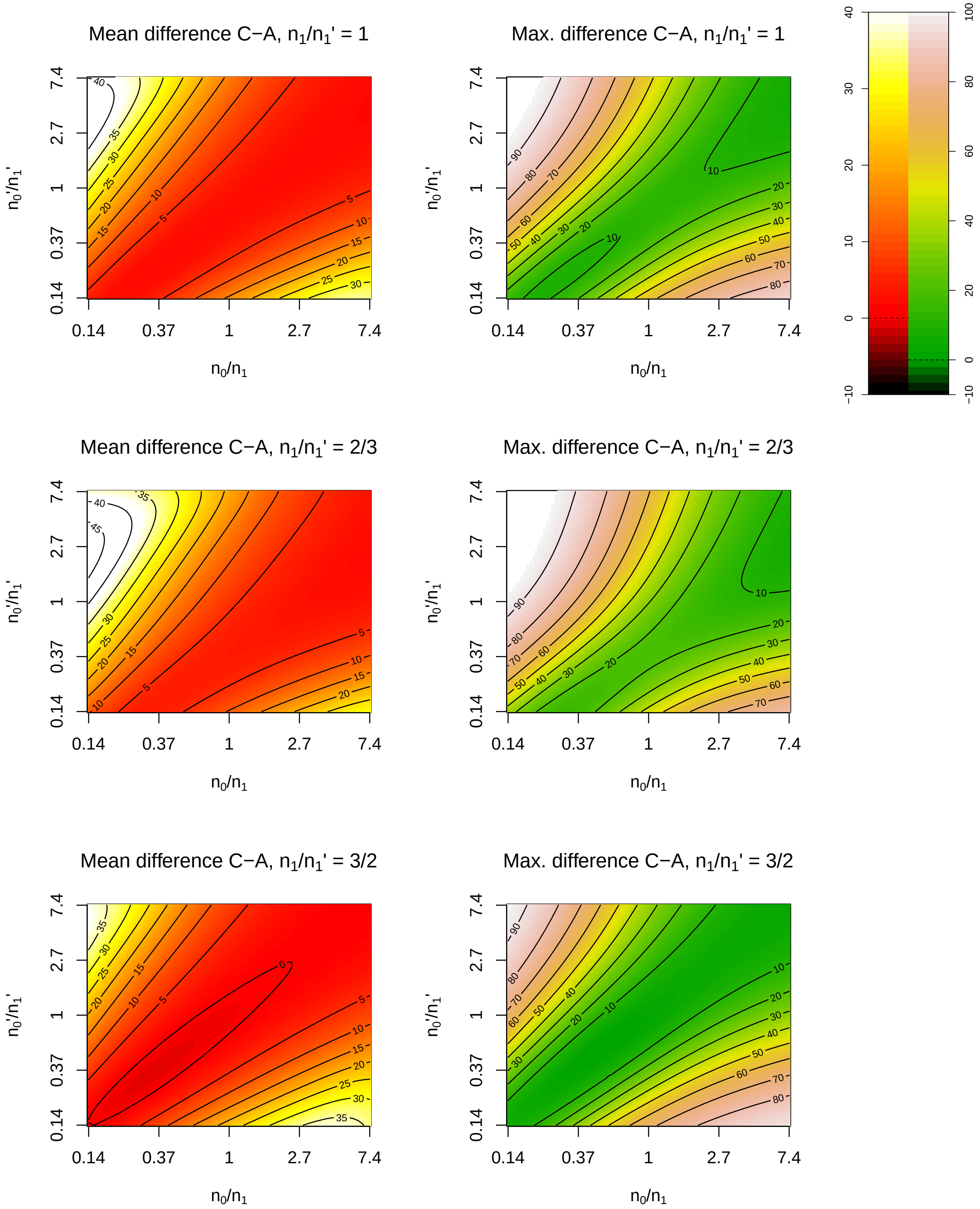

Figure 1: Power difference (%) between methods C and A. Mean power difference is taken as the integral of power difference between methods (see methods section) over $\mathbb{R}$ with respect to log-odds ratio. In all cases, 20 000 samples are used overall for a SNP with MAF 0.1, with cutoffs $\alpha = 5 \times 10^{-6}$, $\beta = 5 \times 10^{-4}$, $\gamma = 5 \times 10^{-8}$. Method C is almost universally more powerful.

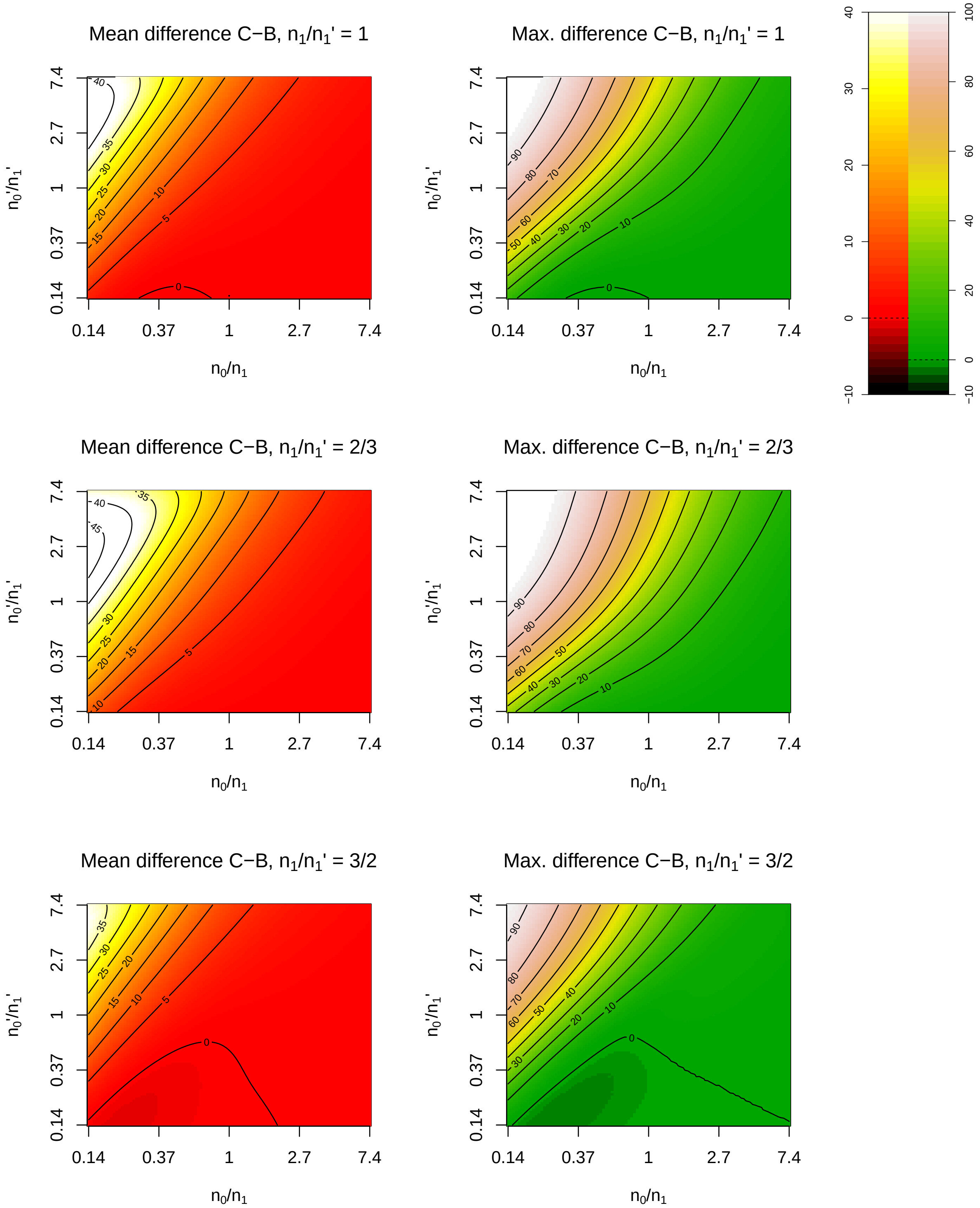

Figure 2: Power difference (%) between methods C and B. Mean power difference is taken as the integral of power difference between methods (see methods section) over $\mathbb{R}$ with respect to log-odds ratio. In all cases, 20 000 samples are used overall for a SNP with MAF 0.1, with cutoffs $\alpha = 5 \times 10^{-6}$, $\beta = 5 \times 10^{-4}$, $\gamma = 5 \times 10^{-8}$.

1 Covariance between Z scores due to shared samples

The matching of type-1 error rates between methods relies on establishing the covariance between triples of z-scores under $H_0^=$. The covariance can be readily approximated when z-scores are assumed to be derived from tests of equality of binomial random variables m_i. Z-scores comparing proportions without using additional information (such as strata or covariates) which are monotonic to allelic difference and asymptotically have $N(0,1)$ distribution must be asymptotically equivalent to those derived from binomial comparisons, so this assumption is reasonable.

If strata or covariates are used, either an assumption must be made that computed z-scores are well-approximated by comparisons of binomial proportions, or correlations must be approximated allowing for the covariate or strata structure. This is sometimes tractable analytically, but can also be estimated empirically either by using known non-associated variants or by simulating variants with the same covariate structure.

The presence of strata or covariates also affects the values ζ_i, and if the effects of covariates are large, the approximations in equations 12 in the main paper may be poor. Values ζ_i can be estimated as functions of allelic differences by simulating variants with the same covariate structure.

1.1 No covariates or stratification

Assume study i and j have n_0^i, n_0^j controls and n_1^i, n_1^j cases respectively, of which n_0^{ij} controls and n_1^{ij} cases are shared between both studies. Let m_0, m_1, m_0', m_1', denote the observed allele frequencies of a SNP in the respective cohort, and $\mu_0, \mu_1, \mu_0', \mu_1'$ the expected allele frequency.

If no strata or covariates are used in the calculation of summary statistics, z scores z_d, z_r, z_s, z_m are asymptotically proportional to the allelic differences $m_1 - m_0$, $m_1' - m_0'$, $m_1' - \frac{m_0'n_0' + m_0n_0}{n_0 + n_0'}$, $\frac{m_1n_1 + m_1'n_1'}{n_1 + n_1'} - \frac{m_0'n_0' + m_0n_0}{n_0 + n_0'}$ respectively, since z scores are monotonic with allelic differences and allelic differences are asymptotically normal. Since m_0, m_1, m_0', m_1' are independent and asymptotically normal the multivariate random variables (z_d, z_r, z_m) and (z_d, z_s, z_m) have multivariate normal distributions.

Suppose we have studies i on n_{0i}, n_{1i} controls and cases and j on n_{0j}, n_{1j} controls and cases, with n_{0ij} and n_{1ij} controls and cases shared between studies respectively. Denote by m_{1ij} the observed

allele frequency at shared cases. Since genotypes are independent at independent samples, we have

$$\text{cov}(m_{1i}, m_{1j}) = \text{cov}\left(\frac{n_{1ij}}{n_{1i}} m_{1ij}, \frac{n_{1ij}}{n_{1j}} m_{1ij}\right) = \frac{n_{1ij}^2}{n_{1i} n_{1j}} \text{var}(m_{1ij}) \tag{1}$$

Now assuming $\mu_{1i} \approx \mu_{0i} \approx \mu_{1j} \approx \mu_{0j} = \mu$ the correlation between the observed allelic differences $m_{1i} - m_{0i}$, $m_{1j} - m_{0j}$ is approximated by

$$\begin{aligned}
\text{cor}(m_{1i} - m_{0i}, m_{1j} - m_{0j}) &= \frac{\text{cov}(m_{1i} - m_{0i}, m_{1j} - m_{0j})}{\sqrt{\text{var}(m_{1i} - m_{0i})\text{var}(m_{1j} - m_{0j})}} \\
&\approx \frac{\text{cov}(m_{1i}, m_{1j}) + \text{cov}(m_{0i}, m_{0j})}{\sqrt{\left(\frac{\mu(1-\mu)}{2n_{1i}} + \frac{\mu(1-\mu)}{2n_{0i}}\right)\left(\frac{\mu_{1j}(1-\mu_{1j})}{2n_{1j}} + \frac{\mu_{0j}(1-\mu_{0j})}{2n_{0j}}\right)}} \\
&\approx \frac{\frac{n_{1ij}^2}{n_{1i}n_{1j}} \frac{\mu(1-\mu)}{2n_{1ij}} + \frac{n_{0ij}^2}{n_{0i}n_{0j}} \frac{\mu(1-\mu)}{2n_{0ij}}}{\sqrt{\left(\frac{\mu_{1i}(1-\mu_{1i})}{2n_{1i}} + \frac{\mu_{0i}(1-\mu_{0i})}{2n_{0i}}\right)\left(\frac{\mu_{1j}(1-\mu_{1j})}{2n_{1j}} + \frac{\mu_{0j}(1-\mu_{0j})}{2n_{0j}}\right)}} \\
&= \frac{n_{0i}n_{0j}n_{1ij} + n_{1i}n_{1j}n_{0ij}}{n_{0i}n_{0j}n_{1i}n_{1j}\sqrt{\frac{1}{n_{0i}} + \frac{1}{n_{1i}}}\sqrt{\frac{1}{n_{0j}} + \frac{1}{n_{1j}}}} \qquad (2)
\end{aligned}$$

This holds under $H_0^=$ (in which $\mu_{1i} = \mu_{0i} = \mu_{1j} = \mu_{0j} = \mu$) and approximately holds in general. Expressions for ρ_{ds}, ρ_{dm}, ρ_{rm} and ρ_{sm} may be derived in terms of n_0, n_1, n_0', and n_1'. Specifically

$$\begin{aligned}
det(\Sigma_A) &= 1 - \rho_{dm}^2 - \rho_{rm}^2 \\
&= \frac{(n_0 n_1' - n_0' n_1)^2}{(n_0 + n_0')(n_1 + n_1')(n_0 + n_1)(n_0' + n_1')} \qquad (3) \\
det(\Sigma_B) &= 1 - \rho_{dm}^2 - \rho_{ds}^2 - \rho_{sm}^2 + 2\rho_{dm}\rho_{ds}\rho_{sm} \\
&= \frac{n_0' n_1^2}{(n_0 + n_1)(n_0 + n_0' + n_1')(n_1 + n_1')} \qquad (4)
\end{aligned}$$

so Σ_A is singular if $\frac{n_0}{n_1} = \frac{n_0'}{n_1'}$, and Σ_B if $n_0' n_1 = 0$.

1.2 Z scores with stratification

If computation of Z scores is performed with correction for strata or covariates, formula 2 will not asymptotically hold and may be a poor approximation to the true covariance. The true covariance can be computed in some cases.

If samples are divided into strata $1, 2, ...s$, and n_{pq}^r, m_{pq}^r, μ_{pq}^r denote the number of samples and

observed and expected minor allele frequencies in cohort p, study q, stratum r respectively, then the z score z_i for study $q = i$ is asymptotically given by

$$z_i = \sum_{r \in 1..s} \alpha_{ir} \left(m^r_{1i} - m^r_{0i} \right) \tag{5}$$

for positive values α_{ir} depending on the values n^r_{pi}. If the Cochran-Mantel-Hanszel test is used, then

$$\alpha_{ir} \propto \frac{n^r_{0i} n^r_{1i}}{n^r_{0i} + n^r_{1i}} \tag{6}$$

Suppose that n^r_{0ij} controls and n^r_{1ij} cases are shared between studies i and j in stratum r. Since the values m^r_{pq} are dependent only within the same values of p and r, the correlation between z_i and z_j under the null hypothesis $\mu^r_{0i} \equiv \mu^r_{1i}, \mu^r_{0i} \equiv \mu^r_{1i}$ is given by

$$\begin{aligned} \text{cor}(z_i, z_j) &= \frac{\sum \alpha_{ir} \alpha_{jr} \text{cov}(m^r_{1i} - m^r_{0i}, m^r_{1j} - m^r_{0j})}{\sqrt{\text{var}(m^r_{1i} - m^r_{0i}) \text{var}(m^r_{1j} - m^r_{0j})}} \\ &\approx \frac{\sum_{r \in 1..s} \alpha \left(\frac{n^r_{0ij}}{n^r_{0i} n^r_{0j}} + \frac{n^r_{1ij}}{n^r_{1i} n^r_{1j}} \right)}{\sqrt{\left(\sum \alpha^2_{ir} \left(\frac{1}{n^r_{0i}} + \frac{1}{n^r_{1i}} \right) \right) \left(\sum \alpha^2_{jr} \left(\frac{1}{n^r_{0j}} + \frac{1}{n^r_{1j}} \right) \right)}} \end{aligned} \tag{7}$$

where all sums are over the values of $r \in 1..s$ for which the relevant values of n^r_{pq} are positive.

1.3 Z scores with covariates

If z scores are computed adjusting for one or more covariates, the estimation of correlation is more difficult.

Assume that in a case population C_1 and a control population C_0 the values of some covariate(s) x have different known distributions f_1, f_0, and that genotypes g at some SNP of interest may vary with x. We will assume the populations are large and that f_1, f_0, and $E(g|x)$ are continuous functions of x.

Let g^k_p denote the genotype of individual k in cohort p ($p \in C_0, C_1$) and x^k_p denote covariate value(s), where g^k_p is an observation of a random variable g. An idealised z-score testing association of g with case/control status should be monotonic with each g^k_p and have expectation 0 if x is independent of case/control status, whatever the form of the function $E(g|x)$. Because individual

genotypes are assumed to be independent between individuals, cross-terms of the form $\prod_i g_i$ should carry no additional information from singleton genotypes. We thus assume that z can thus be decomposed into a weighted linear sum of individual genotypes:

$$z \propto \frac{1}{|C_1|} \sum_{k \in C_1} c_1^k g_1^k - \frac{1}{|C_0|} \sum_{k \in C_0} c_0^k g_0^k \tag{8}$$

where the (positive) values c_1^k, c_0^k depend only on the values $x_1^{\cdot}, x_0^{\cdot}$; that is, not on the relationship between g and x, and the constant of proportionality depends on only on the observed allele frequency. Let function $c_0(x), c_1(x)$ denote the values of c_i corresponding to covariate value(s) x in C_0, C_1.

For a null SNP, $E(g|x)$ is independent of case/control status, but may take any (continuous) form. We have

$$\begin{aligned} 0 = E(z) &\propto E\left(\frac{1}{|C_1|} \sum_{i \in C_1} c_i g_i - \frac{1}{|C_0|} \sum_{i \in C_0} c_i g_i\right) \\ \lim_{|C_0|,|C_1| \to \infty} E(z) &\propto \int c_1(x) f_1(x) E(g|x) dx - \int c_0(x) f_0(x) E(g|x) \\ &\propto \int \left(c_1(x) f_1(x) - c_0(x) f_0(x)\right) E(g|x) dx \end{aligned} \tag{9}$$

From a standard result from the calculus of variations, this implies that

$$c_1(x) f_1(x) - c_0(x) f_0(x) \equiv 0 \implies c_1(x) \propto \frac{f(x)}{f_1(x)}, c_0(x) \propto \frac{f(x)}{f_0(x)} \tag{10}$$

for some function f, so the values c_1^k, c_0^k effectively reweight the contribution of individuals to a common density $f(x)$ across x. The procedure of weighting observation k in this way is analogous to a limiting case of stratification, in which weights are defined by the frequency of stratum r (see above). For a constant allelic difference across the range of x, the best common distribution to 'map to' (that is, the distribution maximising the power to discover an allelic difference) does not depend on the relationship between g and x, and hence the best values of c_i should be constant for all functions $E(g|x)$.

Let z_q denote a z-score for study q; n_{pq}, $f_{pq} = f_{pq}(x)$ and C_{pq} denote the number of samples,

density function of x, and set of samples in cohort p, study q; g^i_{pq} and c^k_{pq} denote the normalised genotype of sample k in cohort p, study q and its coefficient in z_q; n_{0s}, n_{1s}, f_{0s}, f_{1s} and C^s_0, C^s_1 the number of shared controls/cases between studies, the density of x amongst the shared samples, and the sets of shared samples; and f_q the common density function to which cases and controls are weighted in study q (equation 10). Then

$$\text{cov}(z_i, z_j) \approx \frac{\frac{1}{n_{0i}n_{0j}} \sum_{k \in C^s_0} c^k_{0i} c^k_{0j} + \frac{1}{n_{1i}n_{1j}} \sum_{k \in C^s_1} c^k_{1i} c^k_{1j}}{\sqrt{\frac{1}{n^2_{1i}} \sum_{k \in C_{1i}} (c^k_{1i})^2 + \frac{1}{n^2_{0i}} \sum_{k \in C_{0i}} (c^k_{0i})^2} \sqrt{\frac{1}{n^2_{1j}} \sum_{k \in C_{1j}} (c^k_{1j})^2 + \frac{1}{n^2_{0j}} \sum_{k \in C_{0j}} (c^k_{0j})^2}} \tag{11}$$

$$\to \frac{\frac{n_{0s}}{n_{0i}n_{0j}} \int f_{0s}(x) \frac{f_i(x) f_j(x)}{f_{0i}(x) f_{0j}(x)} dx + \frac{n_{1s}}{n_{1i}n_{1j}} \int f_{1s}(x) \frac{f_i(x) f_j(x)}{f_{1i}(x) f_{1j}(x) dx}}{\sqrt{\frac{1}{n_{0i}} \int \frac{f_i(x)^2}{f_{0i}(x)} dx + \frac{1}{n_{1i}} \int \frac{f_i(x)^2}{f_{1i}(x)} dx} \sqrt{\frac{1}{n_{0j}} \int \frac{f_j(x)^2}{f_{0j}(x)} dx + \frac{1}{n_{1j}} \int \frac{f_j(x)^2}{f_{1j}(x)} dx}} \tag{12}$$

with integrals over the domain of x, and the limit as sample sizes tend to infinity while ratios between them remain bounded.

Logistic regression models with continuous covariates can only model simple (generally linear) relationships between c_i and x_i, and property 10 may not hold. If the values c^k_{pq} are known, the correlation can be determined using equation 11. If not, some methods for estimating correlation are outlined below.

1.4 Practical estimation of covariance

Although the asymptotic correlation between z scores may be intractable, as long as the z score permits an expansion of the form 8, the correlation will be nearly invariant with allele frequency and change only minimally for SNPs associated with the covariate.

In practical terms, one method to estimate the correlation between z scores is to simply use the sample correlation at a set of variants presumed to be not associated with the main trait of interest. This approach may be unreliable and have limited power due to the difficulty of identifying such variants

Another option is to permute existing genotypes without permuting covariates, and compute correlation between resultant z scores. This has the disadvantage that it is difficult to permute whilst maintaining potential relationships between genotypes and confounders.

Since the correlation should only depend on the sample sizes and structure of covariate distributions, a more convenient and powerful method is to simply simulate sets of genotypes unassociated

with the trait, but potentially associated with covariates in a range of different ways, and compute correlation between the resultant z scores. Given the shortcomings of standard methods such as logistic regression in fully accounting for covariate effects, this is an advisable procedure in any analysis including covariates.

All results in the main paper which require conditions on sample sizes are only approximate when using studies with stratification or covariates, with the approximation worsening with greater differences in covariate values between groups and lower effective sample sizes.

2 Properties of β^*

2.1 Asymptotic properties of β^*

In this appendix, an asymptotic approximation is established for $\beta^*$and it is shown that $\beta^* > \beta$ for all n_0^i, n_0^j, n_1^i, n_1^j, z_α, z_γ. Define Σ_A and Σ_B as per equations 3 in the main paper, and note that Σ_A and Σ_B only differ in their middle row/column. Further define

$$\Sigma_{dm} = \text{var}\left((z_d\, z_m)^t | H_0^{\cup}\right) = \begin{pmatrix} 1 & \rho_{dm} \\ \rho_{dm} & 1 \end{pmatrix} \tag{13}$$

Let $(z'_\alpha\, z'_\gamma)$ be the point in $\{z_d \geq z_\alpha, z_m \geq z_\gamma\}$ at minimal Mahalanobis distance from the origin with respect to Σ_{dm} (ie, minimal $(z_d\, z_m)\Sigma_{dm}^{-1}(z_d\, z_m)^t$). Then for $z'_\gamma - \rho_{dm} z'_\alpha$ held constant, we have

$$\lim_{z'_\gamma \to\infty / z'_\alpha \to\infty} \frac{\sqrt{|\Sigma_A|}\left((\rho_{ds}\rho_{dm} - \rho_{sm})z'_\gamma + (\rho_{dm}\rho_{sm} - \rho_{ds})z'_\alpha + |\Sigma_{dm}| z_{\beta^*}\right)}{\sqrt{|\Sigma_B|}\left(-\rho_{rm} z'_\gamma + \rho_{dm}\rho_{rm} z'_\alpha + |\Sigma_{dm}| z_\beta\right)} = 1 \tag{14}$$

Specifically, for β^* defined as per equation 5, we have

$$\lim_{\alpha \to 0} \frac{z_{\beta^*}}{\sqrt{1 - \rho_{ds}^2} z_\beta + \rho_{ds} z_\alpha} = 1 \tag{15}$$

and $z_{\beta^*} > \max(z_\beta, \sqrt{1 - \rho_{ds}^2} z_\beta + \rho z_\alpha)$ for all z_α. Firstly the following lemma and corollary are established:

Lemma 1. *Let Σ be a positive definite matrix of dimension N, $\mathbf{x}$ be the vector $(x_1\, x_2 ... x_n)^t$, $\mathbf{A_1}$,*

$\mathbf{A_0}$*, and* $\mathbf{Z} = (z_1\, z_2 ... z_n)^t$ *constant vectors of dimension* N *with* $\mathbf{A_1} \neq \mathbf{A_0} \neq 0$*,* C_0 *a constant, and* R *the (closed) region* $x_1 \geq z_1, x_2 \geq z_2, , , x_N \geq z_N$*.*

Define C *as the (unique) value satisfying*

$$\int_R e^{-\frac{1}{2}\mathbf{x^t}\Sigma^{-1}\mathbf{x}} \left(\Phi(\mathbf{A_1^t x} + C) - \Phi(\mathbf{A_0^t x} + C_0)\right) dx_1 dx_2 ... dx_N = 0 \tag{16}$$

Denote $\mathbf{y} = (y_1\, y_2 ... y_N)$ *as the point in* R *at minimal Mahalanobis distance* $M(\mathbf{y})$ *from the origin with respect to* Σ *(usually,* $\mathbf{y} = \mathbf{Z}$*). Consider all regions* R *for which the corresponding value of* $\mathbf{y}$ *lies on the hyperplane* $\mathbf{A_0^t y} + C_0' = 0$*,* $C_0' \neq C_0$*. Then*

$$\lim_{M(y)\to\infty | \mathbf{A_0^t y} + C_0' = 0} \frac{\mathbf{A_1^t y} + C}{\mathbf{A_0^t y} + C_0} = \lim_{M(y)\to\infty | \mathbf{A_0^t y} + C_0' = 0} \frac{\mathbf{A_1^t y} + C}{C_0 - C_0'} = 1 \tag{17}$$

Proof. The value C is unique since the function $\Phi(\mathbf{A_1^t x} + C)$ is continuous and monotonically increasing in C for all $\mathbf{x}$, and hence so is the integrand (and integral).

We proceed from the formal definition of a limit

$$\forall\, \epsilon > 0\ \exists Y \mid \left(M(y) > Y \implies \left| \frac{\mathbf{A_1^t y} + C}{\mathbf{A_0^t y} + C_0} - 1 \right| < \epsilon \right) \tag{18}$$

Because $\mathbf{A_0^t y} + C_0' = 0$, the right-hand side is equivalent to

$$(1 - \epsilon)(C_0 - C_0') - \mathbf{A_1^t y} \leq C \leq (1 + \epsilon)(C_0 - C_0') - \mathbf{A_1^t y} \tag{19}$$

We will show that there exists Y such that $M(y) > Y$ implies that when C takes values at the endpoints of the interval in the integral 19, the integral 16 takes different signs. Since the integral is increasing in C and must be 0, C must lie in the interval in 19 for $M(y) > Y$.

If C takes the upper value, then at $\mathbf{x} = \mathbf{y}$, the value of the integrand is

$$e^{-\frac{1}{2}M(\mathbf{y})} \left(\Phi((1 + \epsilon)(C_0 - C_0')) - \Phi(C_0 - C_0')\right) \tag{20}$$

the sign of which depends on the sign of $C_0 - C_0'$. We shall assume it is positive (with analogous arguments if it is negative). Because $\epsilon > 0$, point $\mathbf{y}$ does not lie on the hyperplane $(\mathbf{A_1^t} - \mathbf{A_0^t})\mathbf{x} +$

$(1+\epsilon)(C_0 - C_0') - \mathbf{A_1^t y} - C_0 = 0$ (on which the integrand of 16 is 0). The distance from $\mathbf{y}$ to the hyperplane is given by

$$D = \frac{|(\mathbf{A_1^t} - \mathbf{A_0^t})\mathbf{y} + (1+\epsilon)(C_0 - C_0') - \mathbf{A_1^t y} - C_0|}{||\mathbf{A_1^t} - \mathbf{A_0^t}||}$$
$$= \frac{|(1+\epsilon)(C_0 - C_0') - C_0 + C_0'|}{||\mathbf{A_1^t} - \mathbf{A_0^t}||} \tag{21}$$

which is independent of $\mathbf{y}$. Consider a hypersphere centred at $\mathbf{y}$ of radius $d < D$. Each point in the hypersphere can be expressed as $\mathbf{y} + \kappa$ with $|\kappa| \leq d$, so within the hypersphere we have

$$\begin{aligned}\Phi(\mathbf{A_1^t x} + C) - \Phi(\mathbf{A_0^t x} + C_0) &= \Phi(\mathbf{A_1^t}(\mathbf{y} + \kappa) + (1+\epsilon)(C_0 - C_0') - \mathbf{A_1^t y}) \\ &\quad - \Phi(\mathbf{A_0^t}(\mathbf{y} + \kappa) + C_0) \\ &= \Phi\left((1+\epsilon)(C_0 - C_0') + \mathbf{A_1^t}\kappa\right) \\ &\quad + \Phi\left((C_0 - C_0') + \mathbf{A_0^t}\kappa\right) \\ &\geq \Phi\left((1+\epsilon)(C_0 - C_0') + |\mathbf{A_1^t}|d\right) \\ &\quad + \Phi\left((C_0 - C_0') - |\mathbf{A_0^t}|d\right) \end{aligned} \tag{22}$$

Thus d can be chosen independently of $\mathbf{y}$ such that $\Phi(\mathbf{A_1^t x}+C) - \Phi(\mathbf{A_0^t x}+C_0)$ is bounded below in the hypersphere by a constant X also independent of $\mathbf{y}$. The function $\Phi(\mathbf{A_1^t x}+C) - \Phi(\mathbf{A_0^t x}+C_0)$ is obviously bounded by ± 2. Let R' be the intersection of R and the hypersphere. The integral 16 now satisfies

$$\begin{aligned}&\int_R e^{-\frac{1}{2}\mathbf{x^t}\Sigma^{-1}\mathbf{x}} \left(\Phi(\mathbf{A_1^t x} + C) - \Phi(\mathbf{A_0^t x} + C_0)\right) dx_1 dx_2 ... dx_N \\ &= \int_{R'} e^{-\frac{1}{2}\mathbf{x^t}\Sigma^{-1}\mathbf{x}} \left(\Phi(\mathbf{A_1^t x} + C) - \Phi(\mathbf{A_0^t x} + C_0)\right) dx_1 dx_2 ... dx_N \\ &\quad + \int_{R\setminus R'} e^{-\frac{1}{2}\mathbf{x^t}\Sigma^{-1}\mathbf{x}} \left(\Phi(\mathbf{A_1^t x} + C) - \Phi(\mathbf{A_0^t x} + C_0)\right) dx_1 dx_2 ... dx_N \\ &> X \int_{R'} e^{-\frac{1}{2}\mathbf{x^t}\Sigma^{-1}\mathbf{x}} dx_1 dx_2 ... dx_N \\ &\quad - 2 \int_{R\setminus R'} e^{-\frac{1}{2}\mathbf{x^t}\Sigma^{-1}\mathbf{x}} dx_1 dx_2 ... dx_N \end{aligned} \tag{23}$$

Because d (the radius of the hypersphere) does not depend on $\mathbf{y}$, by the properties of the Gaussian

integral a value M_+ can be chosen such that $M(y) > M_+$ implies that the ratio

$$\frac{\int_{R'} e^{-\frac{1}{2}\mathbf{x}^{\mathbf{t}}\Sigma^{-1}\mathbf{x}} dx_1 dx_2 ... dx_N}{\int_{R \setminus R'} e^{-\frac{1}{2}\mathbf{x}^{\mathbf{t}}\Sigma^{-1}\mathbf{x}} dx_1 dx_2 ... dx_N} \tag{24}$$

is arbitrarily large (namely, $> 2/X$), and hence integral 23 is positive. In a similar way, a value M_- can be chosen such that if C takes the lower value of interval 19, the integral is negative for $M(y) > M_-$. For $M(y) > \max(M_+, M_-)$, the value of C satisfying equation 16 lies within the interval 19, and the limit is established.

□

Corollary 1. *Given $b, c, y \in \mathbb{R}^+$, define a such that*

$$\int_y^\infty e^{-\frac{x^2}{2}} \left(\Phi(c) - \Phi(a - bx)\right) dx = 0 \tag{25}$$

then

$$\lim_{y \to \infty} \frac{a}{by + c} = 1 \tag{26}$$

and $a > by + c \; \forall \, y$

Proof. We note firstly that the function $\Phi(c) - \Phi(a - bx)$ is increasing for all x. If the integral is 0, the (smooth) integrand must cross 0 at some finite $x \in (y, \infty)$, and hence its value at $x = y$ must be negative. As Φ is increasing, we have $\Phi(a - by) > \Phi(c) \implies a > by + c$

The proof of the limit proceeds in a similar way to the proof of the lemma above.

□

Now (recalling definition 4 in the main paper)

$$\begin{aligned}
&\int_{z_\alpha}^\infty \int_{z_\gamma}^\infty \int_{z_{\beta*}}^\infty N_{\Sigma_B}\left((z_d\, z_s\, z_m)^t\right) dz_s dz_m dz_d \\
&= \int_{z_\alpha}^\infty \int_{z_\gamma}^\infty \int_{z_\beta}^\infty N_{\Sigma_A}\left((z_d\, z_r\, z_m)^t\right) dz_r dz_m dz_d \\
&\implies \int_{z_\alpha}^\infty \int_{z_\gamma}^\infty N_{\Sigma_{dm}}\left((z_d\, z_m)^t\right) \left(\Phi\left(a_1 z_d + b_1 z_m + c_1\right)\right. \\
&\qquad\qquad \left. -\Phi\left(a_0 z_d + b_0 z_m + c_0\right)\right) dz_d dz_m = 0
\end{aligned} \tag{27}$$

where

$$
\begin{aligned}
a_0 &= -\frac{\rho_{dm}\rho_{rm}}{\sqrt{|\Sigma_{dm}||\Sigma_A|}} \\
b_0 &= \frac{\rho_{rm}}{\sqrt{|\Sigma_{dm}||\Sigma_A|}} \\
c_0 &= -\sqrt{\frac{|\Sigma_{dm}|}{|\Sigma_A|}} z_\beta \\
a_1 &= \frac{\rho_{ds} - \rho_{dm}\rho_{sm}}{\sqrt{|\Sigma_{dm}||\Sigma_B|}} \\
b_1 &= \frac{\rho_{sm} - \rho_{ds}\rho_{dm}}{\sqrt{|\Sigma_{dm}||\Sigma_B|}} \\
c_0 &= -\sqrt{\frac{|\Sigma_{dm}|}{|\Sigma_B|}} z_{\beta^*} \qquad (28)
\end{aligned}
$$

The asymptotic property of β^* follows from corollary 1.

If $\gamma = 1$, we have from definition 5 in the main paper

$$
\begin{aligned}
&\int_{z_\alpha}^{\infty}\int_{z_{\beta^*}}^{\infty} \frac{1}{2\pi\sqrt{1-\rho_{ds}^2}} \exp\left(-\frac{1}{2(1-\rho_{ds}^2)}\left(x^2+y^2-2\rho xy\right)\right) dxdy \\
&= \int_{z_\alpha}^{\infty}\int_{z_\beta}^{\infty} \frac{1}{2\pi} \exp\left(-\frac{1}{2}\left(x^2+y^2\right)\right) dxdy \\
&\implies \int_{z_\alpha}^{\infty} e^{-\frac{y^2}{2}} \Phi\left(\frac{z_{\beta^*} - \rho_{ds} y}{\sqrt{1-\rho_{ds}^2}}\right) dy = \int_{z_\alpha}^{\infty} e^{-\frac{y^2}{2}} \Phi(z_\beta) \qquad (29)
\end{aligned}
$$

from which the result follows from an application of lemma 1.

2.2 Size of β, β^* and $\beta^\perp$

To show that $\beta^* < \beta$, we show that if we set $z_{\beta^*} = z_\beta$ in the integral 27, then the integral is positive. Since it is decreasing with z_β^* (as Φ is increasing) we must have $z_\beta^* > z_\beta$ if the integral is to be 0. A similar argument can be used to show that $\beta^\perp < \beta^*$. Denote by $I(z_d, z_m)$ the value of the integrand of 27 with $z_{\beta^*} = z_\beta$.

Consider the line $a_1 z_d + b_1 z_m + c_1 = a_0 z_d + b_0 z_m + c_0$ on the (z_d, z_m) plane on which the integrand

of 27 is 0. The gradient of this line is

$$\frac{a_0 - a_1}{b_0 - b_1} = \frac{\sqrt{n_0^i(n_0 + n_0')n_1(n_0 + n_1)}}{\sqrt{(n_1 + n_1')(n_0 + n_0' + n_1 + n_1')}} \times \tag{30}$$

$$\frac{n_0' n_1(n_0 + n_0' + n_1 + n_1') - (n_0 + n_0')|n_0' n_1 - n_0 n_1'|}{n_0'(n_0 + n_0')n_1(n_0 + n_1) - (n_0^2 + n_0 n_0' + n_0' n_1)|n_0' n_1 - n_0 n_1'|} \tag{31}$$

Since $|n_0' n_1 - n_0 n_1'| \geq (n_0' n_1 - n_0 n_1')$ the numerator of the second fraction is greater than or equal to

$$\begin{aligned} n_0' n_1(n_0 + n_0' + n_1 + n_1') - (n_0 + n_0')(n_0' n_1 - n_0 n_1') &= n_0^2 n_1' + n_0'(n_1^2 + n_0 n_1' + n_1 n_1') \\ &> 0 \end{aligned} \tag{32}$$

and similarly the denominator is greater than or equal to

$$n_0(n_0^2 n_1' + n_0'(n_1^2 + n_0 n_1' + n_1 n_1')) > 0 \tag{33}$$

so the gradient is positive. If $b_1 - b_0 > 0$, $I(z_d, z_m)$ is positive if (z_d, z_m) falls above the line, and negative if below it; if $b_1 - b_0 < 0$, the other way around. Assume for the moment that $b_1 - b_0 < 0$.

If the point (z_α, z_γ) lies above the line, then since $I(z_d, z_m)$ is negative in the region $(-\infty, z_\alpha) \times (z_\gamma, \infty)$, we have

$$\begin{aligned} \int_{z_\alpha}^{\infty} \int_{z_\gamma}^{\infty} I(z_d, z_m) dz_d dz_m &\geq \int_{z_\alpha}^{\infty} \int_{z_\gamma}^{\infty} I(z_d, z_m) dz_d dz_m \\ &\quad + \int_{-\infty}^{z_\alpha} \int_{z_\gamma}^{\infty} I(z_d, z_m) dz_d dz_m \\ &= \int_{\infty}^{\infty} \int_{z_\gamma}^{\infty} I(z_d, z_m) dz_d dz_m \end{aligned} \tag{34}$$

If the point lies below the line, let $z_\gamma' > z_\gamma$ be defined such that the point (z_α, z_γ') lies on the line. Since $I(z_d, z_m)$ is positive in the region $(z_\alpha, \infty) \times (z_\gamma, z_\gamma')$ and negative in the region $(-\infty, z_\alpha) \times$

(z_γ, ∞), we have

$$\begin{aligned}\int_{z_\alpha}^{\infty}\int_{z_\gamma}^{\infty} I(z_d, z_m) dz_d dz_m &\geq \int_{z_\alpha}^{\infty}\int_{z_\gamma}^{\infty} I(z_d, z_m) dz_d dz_m \\ &- \int_{z_\alpha}^{\infty}\int_{z_\gamma}^{z'_\gamma} I(z_d, z_m) dz_d dz_m \\ &+ \int_{-\infty}^{z_\alpha}\int_{z'_\gamma}^{\infty} I(z_d, z_m) dz_d dz_m \\ &= \int_{-\infty}^{\infty}\int_{z'_\gamma}^{\infty} I(z_d, z_m) dz_d dz_m \end{aligned} \tag{35}$$

so it is sufficient to prove that the integral is positive when the range (z_α, ∞) is replaced with $(-\infty, \infty)$. Similar arguments can be used when $b_1 - b_0 > 0$, in which case it is sufficient to prove positivity when $z_\gamma = 0$.

This enables z_d (or z_m) to be integrated out, namely reducing to showing that

$$\begin{aligned} &\int_{z_\beta}^{\infty}\int_{z_\gamma}^{\infty} N_{\begin{pmatrix} 1 & \rho_{sm} \\ \rho_{sm} & 1 \end{pmatrix}}((z_s\, z_m)^t) - N_{\begin{pmatrix} 1 & \rho_{rm} \\ \rho_{rm} & 1 \end{pmatrix}}((z_s\, z_m)^t) dz_m dz_s > 0 \\ \Leftrightarrow &\int_{z_\beta}^{\infty} \frac{1}{2\pi} \exp\left(\frac{1}{2} z_s^2\right)\left(\Phi\left(\frac{\rho_{sm} z_s - z_\gamma}{1-\rho_{sm}^2}\right) - \Phi\left(\frac{\rho_{rm} z_s - z_\gamma}{1-\rho_{rm}^2}\right)\right) > 0 \end{aligned} \tag{36}$$

The second part of the integrand is monotonically increasing in z_s as $\rho_{sm} > \rho_{rm}$. Thus the integral is minimised as $z_\beta \to -\infty$, at which the value is $\Phi(z_\gamma)$, which is positive.

3 SNPs with aberrant allele frequency in one group

3.1 $R_B < R_A$ for SNPs with aberrance in C_1

If SNPs have aberrant MAF in C_1 only, we have $E(z_d) = \zeta_d \neq 0$, $E(z_m) = \zeta_m \neq 0$ and $E(z_s) = E(z_r) = 0$. As noted in the main text, as $\zeta_d \to 0$, $R_B, R_A \to P_0$ (equation 4 in the main paper) and

$$\begin{aligned}\lim_{\zeta_d \to \infty} R_B = \lim_{\zeta_d \to \infty} &\left(\int_{z_\alpha - \zeta_d}^{\infty}\int_{z_{\beta^*}}^{\infty}\int_{z_\gamma - \zeta_m}^{\infty} N_{\Sigma_B}\left((z_d\, z_s\, z_m)^t\right) dz_s dz_m dz_d \right. \\ &\left. + \int_{z_\alpha + \zeta_d}^{\infty}\int_{z_{\beta^*}}^{\infty}\int_{z_\gamma + \zeta_m}^{\infty} N_{\Sigma_B}\left((z_d\, z_s\, z_m)^t\right) dz_s dz_m dz_d\right) \\ = \Phi(-z_\beta^*) = \frac{\beta^*}{2} \end{aligned} \tag{37}$$

and similarly, $R_A \to \frac{\beta}{2}$, $R_B \to \frac{\beta^*}{2}$ as $\zeta_d \to \pm\infty$, with $\beta^* < \beta$ as shown above. For β^* defined by 5 in the main paper, we show here that $R_A > R_B$ for all ζ_d. For the more general definition of β^* (equation 4 in the main paper), the inequality $R_B < R_A$ may not hold for all ζ_d. However, in practice, the inequality holds for almost all ζ_d and any deviation is small and near $\zeta_d = 0$.

Define the shorthand $N_\rho(x, y)$ as the value at (x, y) of the bivariate normal PDF with mean $\binom{0}{0}$ and variance $\begin{pmatrix} 1 & \rho \\ \rho & 1 \end{pmatrix}$, and $\text{erfc}(x) = 2\left(1 - \Phi(\sqrt{2}x)\right)$ as the complementary error function. In this section, ρ refers to ρ_{ds}.

Consider the value $R_A - R_B$ as a function of ζ_d. We will show that the partial derivative $\frac{\delta}{\delta\zeta_d}(R_A - R_B)$ is zero only when $\zeta_d = 0$. Because $R_A - R_B$ passes through the origin and is asymptotically positive in both directions, it is positive for all $\zeta_d \neq 0$. We have

$$\begin{aligned} R_A - R_B = &\left(\int_{z_\beta}^{\infty}\int_{z_\alpha-\zeta_d}^{\infty} N_0(x,y)dxdy - \int_{z_{\beta^*}}^{\infty}\int_{z_\alpha-\zeta_d}^{\infty} N_\rho(x,y)dxdy\right) \\ &+ \left(\int_{-\infty}^{-z_\beta}\int_{-\infty}^{-z_\alpha+\zeta_d} N_0(x,y)dxdy - \int_{-\infty}^{-z_{\beta^*}}\int_{-\infty}^{-z_\alpha+\zeta_d} N_\rho(x,y)dxdy\right) \end{aligned} \quad (38)$$

$$\begin{aligned} \frac{\delta}{\delta\zeta_d}(R_A - R_B) = &\left(\int_{z_\beta}^{\infty}\frac{\delta}{\delta\zeta_d}\int_{z_\alpha-\zeta_d}^{\infty} N_0(x,y)dxdy - \int_{z_{\beta^*}}^{\infty}\frac{\delta}{\delta\zeta_d}\int_{z_\alpha-\zeta_d}^{\infty} N_\rho(x,y)dxdy\right) \\ &+ \left(\int_{z_\beta}^{\infty}\frac{\delta}{\delta\zeta_d}\int_{z_\alpha+\zeta_d}^{\infty} N_0(x,y)dxdy - \int_{z_{\beta^*}}^{\infty}\frac{\delta}{\delta\zeta_d}\int_{z_\alpha+\zeta_d}^{\infty} N_\rho(x,y)dxdy\right) \\ = &\frac{1}{2\sqrt{2\pi}}\text{erfc}\left(\frac{z_\beta}{\sqrt{2}}\right)\left(e^{-\frac{1}{2}(\zeta_d-z_\alpha)^2} - e^{-\frac{1}{2}(\zeta_d+z_\alpha)^2}\right) \\ -\frac{1}{2\sqrt{2\pi}}&\left(e^{-\frac{1}{2}(\zeta_d-z_\alpha)^2}\text{erfc}\left(\frac{z_{\beta^*}+\rho(\zeta_d-z_\alpha)}{\sqrt{2(1-\rho^2)}}\right) - e^{-\frac{1}{2}(\zeta_d+z_\alpha)^2}\text{erfc}\left(\frac{z_{\beta^*}-\rho(\zeta_d+z_\alpha)}{\sqrt{2(1-\rho^2)}}\right)\right) \end{aligned}$$

Showing that $\frac{\delta}{\delta\zeta_d}(R_A - R_B) > 0$ when $\zeta_d > 0$ is equivalent to showing that $(a - b) - (pa - qb) > 0$ where $a = e^{-\frac{1}{2}(\zeta_d-z_\alpha)^2}$, $b = e^{-\frac{1}{2}(\zeta_d+z_\alpha)^2}$, $p = \frac{\text{erfc}\left(\frac{z_{\beta^*}+\rho(\zeta_d-z_\alpha)}{\sqrt{2(1-\rho^2)}}\right)}{\text{erfc}\left(\frac{z_\beta}{2}\right)}$ and $q = \frac{\text{erfc}\left(\frac{z_{\beta^*}+\rho(\zeta_d-z_\alpha)}{\sqrt{2(1-\rho^2)}}\right)}{\text{erfc}\left(\frac{z_\beta}{2}\right)}$.

Since $(\zeta_d - z_a)^2 < (\zeta_d + z_a)^2$ for $\zeta_d > 0$, we have $a > b$. Because erfc is strictly decreasing, we have $p < q$. Because $\frac{\delta p}{\delta\zeta_d} < 0$, we have

$$p < \frac{\text{erfc}\left(\frac{z_{\beta^*}-z_\alpha)}{\sqrt{2(1-\rho^2)}}\right)}{\text{erfc}\left(\frac{z_\beta}{2}\right)} < 1 \quad (39)$$

where the second inequality arises because $z_{\beta^*} > \sqrt{1-\rho^2}z_\beta + \rho z_\alpha$. Thus $pa - qb < pa - pb =$

$p(a-b) < a-b$, and $\frac{\delta}{\delta\zeta_d}(R_A - R_B) > 0$ as required.

4 Upper bound on $R_B - R_A$ with aberrance in C_1'

For SNPs with aberrance in C_1', we have $E(z_d) = 0$, $E(z_r) = \zeta_r \neq 0$, $E(z_s) = \zeta_s \neq 0$ and $E(z_m) = \zeta_m \neq 0$. As above $R_A, R_B \to P_0$ as $\zeta_r \to 0$, and by similar arguments to the section above, $R_A, R_B \to \frac{\alpha}{2}$ as $\zeta_r \to \pm\infty$.

For β^* defined as per equation 5 in the main paper, it is possible to derive an approximate (asymptotically accurate) upper bound on $R_B - R_A$, corresponding to the most serious increase in FPR. The approach is not readily applied to the general definition of β^*, but again the difference is typically small in practice.

To first order

$$\frac{\zeta_s}{\zeta_r} = \frac{\left(\frac{\mu_1' - \mu_0'}{SE(m_1' - m_0')}\right)}{\left(\frac{\mu_1' - \mu_0}{SE\left(m_1' - \frac{m_0 n_0 + m_0' n_0'}{n_0 + n_0'}\right)}\right)} = \sqrt{\frac{(n_0 + n_0')(n_0' + n_1'}{n_0'(n_0 + n_0' + n_1')}} \stackrel{\text{def}}{=} k \tag{40}$$

Now

$$\begin{aligned} R_B - R_A = &\left(\int_{z_{\beta^*} - \zeta_s}^{\infty} \int_{z_\alpha}^{\infty} N_\rho(x,y) dx dy - \int_{z_\beta - \zeta_r}^{\infty} \int_{z_\alpha}^{\infty} N_0(x,y) dx dy\right) \\ &+ \left(\int_{z_{\beta^*} + \zeta_s}^{\infty} \int_{z_\alpha}^{\infty} N_\rho(x,y) dx dy - \int_{z_\beta + \zeta_r}^{\infty} \int_{z_\alpha}^{\infty} N_0(x,y) dx dy\right) \end{aligned} \tag{41}$$

Define z_r^+, z_r^- such that

$$\begin{aligned} \int_{z_r^-}^{\infty} \int_{z_\alpha}^{\infty} N_0(x,y) dx dy &= \int_{z_{\beta^*} - \zeta_s}^{\infty} \int_{z_\alpha}^{\infty} N_\rho(x,y) dx dy \\ \int_{z_r^+}^{\infty} \int_{z_\alpha}^{\infty} N_0(x,y) dx dy &= \int_{z_{\beta^*} + \zeta_s}^{\infty} \int_{z_\alpha}^{\infty} N_\rho(x,y) dx dy \end{aligned} \tag{42}$$

From equation 15 in Appendix 2.1, we have $z_{\beta^*} - \zeta_s \approx \sqrt{1-\rho^2} z_r^- - \rho z_\alpha$ and $z_{\beta^*} + \zeta_s \approx \sqrt{1-\rho^2} z_r^+ - \rho z_\alpha$.

Noting that $\int_a^\infty \int_b^\infty N_0(x,y)dxdy = \Phi(-a)\Phi(-b)$ and $\Phi(x) = 1 - \Phi(-x)$ we now have

$$R_B - R_A = \Phi(-z_\alpha)\left(\Phi(z_\beta - \zeta_r) - \Phi(z_r^-) + \Phi(z_\beta + \zeta_r) - \Phi(z_r^+)\right) \tag{43}$$

Applying the identity $\Phi(-z_\alpha) = \frac{\alpha}{2}$ and approximations $z_\beta^* \approx \sqrt{1-\rho^2} z_\beta + \rho z_\alpha$, $\zeta_s \approx k\zeta_r$, yields

$$\begin{aligned} z_r^- &\approx \frac{z_{\beta^*} - \zeta_s + \rho z_\alpha}{\sqrt{1-\rho^2}} \approx z_\beta - \frac{k}{\sqrt{1-\rho^2}}\zeta_r \\ z_r^+ &\approx z_\beta + \frac{k_1}{\sqrt{1-\rho^2}} z_0' \end{aligned} \tag{44}$$

$$R_B - R_A \approx \frac{\alpha}{2}\left(\Phi\left(z_\beta - \frac{k}{\sqrt{1-\rho^2}}\zeta_r\right) - \Phi(z_\beta - \zeta_r) + \Phi\left(z_\beta + \frac{k}{\sqrt{1-\rho^2}}\zeta_r\right) - \Phi(z_\beta + \zeta_r)\right) \tag{45}$$

Considered as a function of ζ_r, the value $R_B - R_A$ will be 0 at $\zeta_r = 0$ and tend to 0 as $\zeta_r \to \pm\infty$. It will be maximised approximately at the points where $\Phi(z_\beta - \zeta_r)$ or $\Phi(z_\beta + \zeta_r)$ are changing most rapidly; that is, $\zeta_r = \pm z_\beta$. At $\zeta_r = z_\beta$, the contribution to the value $R_B - R_A$ from the difference $\Phi\left(z_\beta + \frac{k}{\sqrt{1-\rho^2}}\zeta_r\right) - \Phi(z_\beta + \zeta_r)$ is negligible (and similarly for the other difference when $\zeta_r = -z_\beta$). Using the first-order approximation for $\Phi(z_\beta - \zeta_r)$ about $\zeta_r = z_\beta$ yields

$$\max(R_B - R_A) \approx \frac{\alpha}{2\sqrt{2\pi}}\left(\frac{k}{\sqrt{1-\rho^2}} - 1\right) z_\beta \tag{46}$$

In general, this value is substantially less than α.

All instances of 'approximately equal' are asymptotic limits as $z_a \to \infty$ and n_0, $n_{0'}$, n_1, $n_1' \to \infty$ with z_0' held finite.

5 Aberrance in C_0'

For SNPs aberrant in C_0', again $E(z_d) = 0$, $E(z_r) = \zeta_r \neq 0$, $E(z_s) = \zeta_s \neq 0$ and $E(z_m) = \zeta_m \neq 0$. As above $R_A, R_B \to P_0$ as $\zeta_r \to 0$, and $R_A, R_B \to \frac{\alpha}{2}$ as $\zeta_r \to \pm\infty$. In this case, R_B is typically less than R_A.

6 General aberrance in replication cohorts

For β^* defined according to 5 in the main paper, the increase in FPR $R_B - R_A$ for method B for a SNP with aberrance in C_1' is generally smaller than the decrease in FPR $R_A - R_B$ for a SNP with a similarly-sized aberrance in C_0', in that the integral of the difference over the range of ζ_r is generally smaller in the former.

We define k as in the section above and $k_1 = \frac{\zeta_s}{\zeta_r}\Big|C_0' \text{ aberrant} = \sqrt{\frac{n_0'(n_0'+n_1')}{(n_0+n_0')(n_0+n_0'+n_1')}}$ similarly. Now for C_0' aberrant

$$R_A - R_B \approx \frac{\alpha}{2}\left(\Phi(z_\beta - \zeta_r) - \Phi\left(z_\beta - \frac{k_1}{\sqrt{1-\rho^2}}\zeta_r\right) + \Phi(z_\beta + \zeta_r) - \Phi\left(z_\beta + \frac{k_1}{\sqrt{1-\rho^2}}\zeta_r\right)\right) \quad (47)$$

Since $\int_0^x \Phi(z)dz = x\Phi(x) + \frac{1}{\sqrt{2\pi}}\left(e^{-\frac{x^2}{2}} - 1\right)$, we have

$$\int_0^\infty \left(\Phi(h-z) - \Phi(h-kz)\right)dz = \left(1 - \frac{1}{k}\right)\left(\frac{1}{\sqrt{2\pi}}e^{-\frac{1}{2}h^2} + h\Phi(h)\right) \quad (48)$$

$$\int_0^\infty \left(\Phi(h+z) - \Phi(h+kz)\right)dz = \left(1 - \frac{1}{k}\right)\left(-\frac{1}{\sqrt{2\pi}}e^{-\frac{1}{2}h^2} + h\Phi(-h)\right) \quad (49)$$

Thus with aberrant C_0'

$$\int_0^\infty (R_A - R_B)\,d\zeta_r = \frac{\alpha}{2}\left(1 - \frac{\sqrt{1-\rho^2}}{k_1}\right)z_\beta \quad (50)$$

Comparing R_A and R_B under the two aberrance scenarios with the same ζ_d

$$\frac{\int_0^\infty (R_A - R_B)\,d\zeta_d\ [C_0' \text{ aberrant}]}{\int_0^\infty (R_B - R_A)\,d\zeta_d\ [C_1' \text{ aberrant}]} = \frac{1 - \frac{\sqrt{1-\rho^2}}{k_1}}{\frac{\sqrt{1-\rho^2}}{k} - 1} \quad (51)$$

For this to be > 1, a necessary condition is $\left(1 - \frac{\sqrt{1-\rho^2}}{k_2}\right) > \left(\frac{\sqrt{1-\rho^2}}{k_1} - 1\right)$ From the definitions of ρ_{ds} (Appendix 1), k (equation 40) and k_1, this is equivalent to

$$\sqrt{\frac{n_0 + n_0' + n_1'}{n_0' + n_1'}}\sqrt{1 - \frac{n_0 n_1 n_1'}{(n_0+n_0')(n_0+n_1)(n_0+n_0'+n_1')}}\left(\sqrt{\frac{n_0'}{n_0+n_0'}} + \sqrt{\frac{n_0+n_0'}{n_0}}\right) > 2$$

The final term in this product is of the form $x + \frac{1}{x}$ so is greater than 2. A sufficient condition is

thus

$$\frac{n_0+n_0'+n_1'}{n_0'+n_1'}\left(1-\frac{n_0 n_1 n_1'}{(n_0+n_0')(n_0+n_1)(n_0+n_0'+n_1')}\right)\geq 1$$
$$\iff n_0^2+n_0(n_0'+n_1)+n_1(n_0'-n_1')\geq 0 \tag{52}$$

which holds in most study designs.
